# Intuitionistic fuzzy fairly operators and additive ratio assessment-based integrated model for selecting the optimal sustainable industrial building options

**DOI:** 10.1038/s41598-023-31843-x

**Published:** 2023-03-28

**Authors:** Arunodaya Raj Mishra, Pratibha Rani, Fausto Cavallaro, Ibrahim M. Hezam

**Affiliations:** 1 Department of Mathematics, Government College Raigaon, Satna, Madhya Pradesh 485441 India; 2 Department of Engineering Mathematics, Koneru Lakshmaiah Education Foundation, Guntur, Andhra Pradesh 522302 India; 3 Department of Economics, University of Molise, Via De Sanctis, 86100 Campobasso, Italy; 4 Department of Statistics and Operations Research, College of Sciences, King Saud University, Riyadh, Saudi Arabia

**Keywords:** Computational science, Environmental impact

## Abstract

In the past few years, the private sectors and industries have focused their attention on sustainable development goals to achieve the better and more sustainable future for all. To accomplish a sustainable community, one requires to better recognize the fundamental indicators and selects the most suitable sustainable policies in diverse regions of the community. Considering the huge impact of construction industry on sustainable development, very less research efforts have been made to obtain worldwide sustainable elucidations for this type of industry. As a large sector of construction industry, industrial buildings consume enormous amounts of energy and financial assets, and play a key character in job creation and life quality improvement in the community. In order to assess the sustainable industrial buildings by means of multiple indicators, the present study introduces a hybrid multi-criteria decision-making methodology which integrates the fairly aggregation operator, the MEthod based on the Removal Effects of Criteria (MEREC), the stepwise weight assessment ratio analysis (SWARA) and the additive ratio assessment (ARAS) methods with intuitionistic fuzzy set (IFS). In this respect, firstly new intuitionistic fuzzy weighted fairly aggregation operators are proposed and then employed to aggregate the decision information in the proposed hybrid method. This operator overcomes the limitations of basic intuitionistic fuzzy aggregation operators. To find the criteria weights, an integrated model is presented based on the MEREC for objective weights and the SWARA for subjective weights of indicators under IFS context. To rank the sustainable industrial buildings, an integrated ARAS method is employed from uncertain perspective. Further, a case study concerning sustainable industrial buildings evaluation is presented to illustrate the superiority and practicality of the developed methodology. The advantages of the developed approach are highlighted in terms of stability and reliability by comparison with some of the existing methods.

## Introduction

“Sustainable development (SD)” aims to minimize the ecological footprints of human activities on the environment while ensuring socio-economic development. The concept of SD has been defined in a different way by diverse organizations and sectors^1^. Construction industry is normally one of the leading businesses in both developed and developing nations in respect of employment, involvement to “gross domestic product (GDP)”, environmental footprint and investment^2,3^. As one of the prime consumers of natural resources, the construction industry has a big part to play in SD. The effective management of the construction industry results in boosted tourism, improved life quality, money circulation, sustainable environment and job creation throughout the country^4^.

“Industrial buildings (IBs)”, one of the largest segments of construction industry, are factories or other large premises mainly used for storing or manufacturing raw materials, goods or services for economic purposes^5,6^. Despite of significant role of IBs in SD, few studies have been conducted to establish IBs from sustainable perspective. For instance, Zhao et al.^7^ employed the data envelopment analysis for prioritizing the most sustainable large development projects. In a study, Zeng et al.^8^ put forward hierarchy cluster analysis for investigating industrial sustainability of the manufacturing region. A novel automated tool has been developed for evaluating economic and environmental indicators in the simulation process of sour water stripping plant^9^. To achieve sustainable development goals in Iran, Heravi et al.^10^ assessed the IBs with sustainable viewpoints. Heravi et al.^11^ studied a new decision-making methodology by combining grey doctrine and utility degree with ELECTRE method for assessing “sustainable industrial buildings (SIBs)”.

In earlier times, IB was considered as an isolated container under which production activities took place^6^. Nowadays, the design of IBs is not only limited to four walls and a roof in which certain production activities occurs but it considers the sustainable aspects including contamination caused by the construction process, reduction of greenhouse gas emissions, waste disposal, recycling, workers’ safety, job creation, minimum economic cost etc. With several conflicting qualitative and quantitative sustainability indicators/criteria, the process of SIB selection can be treated as a “multi-criteria decision-making (MCDM)” problem. Due to increasing complexity, imprecise data and vagueness of human mind, it is difficult for the “decision-making experts (DMEs)” to present exact numerical values for the considered attributes. In this regard, Zadeh’s proposal of “fuzzy set theory (FST)” has been accepted as a valuable tool and widely used in implementation to address the ambiguity of human decision.

Further, Atanassov^12^ gave the theory of “intuitionistic fuzzy set (IFS)” to get over certain limitations of Zadeh’s FST. It is characterized by the “belongingness degree (BD)” and “non-belongingness degree (ND)”, wherein the values of BD and ND are real numbers between 0 and 1. The main difference between FST and IFS lies in the form of the expression: for FST, only the degree of belongingness is given to each element of the universe,while for IFS, not only the degree of belongingness but also the degree of non-belongingness is given, and their sum is less than or equal to one. In comparison with FST, IFSs could reflect individual’s evaluation results from both positive and negative aspects. There are several situations that can be modelled using IFS but cannot be represented using classical FST. For example, suppose voters may be partitioned into three groups of those who vote for, who vote against and who abstain. If we take $\left\langle {v_{1} ,\,\,0.7,\,\,0.2} \right\rangle$ as an element of IFS *L* of voting, we can interpret that “the vote for the applicant is 0.7 in favor to 0.2 against with 0.1 nonparticipations”. Therefore, IFSs are more comprehensive and reasonable than classical fuzzy sets in describing the uncertainty of an object. After the pioneering work of Atanassov^12^, several researchers have presented many theories that have been widely used in the fields of clustering, pattern recognition, matching problem, plant leaf recognition, stock prediction etc^13–15^.

### Research gaps

Based on the prior researches, the following challenges are identified:

- In the literature, some articles^8,10,16–22^ have presented to assess the sustainability in IBs but there is no study which considers the uncertainty of SIBs from intuitionistic fuzzy perspective.
- Several MCDM methods^23–34^ have been developed under intuitionistic fuzzy environment, but the “aggregation operators (AOs)” used in these studies have some counter-intuitive cases.
- The sustainability indicators play an important role in the assessment of IBs. However, the criteria weighting models given by^10,17,19,22^ are suitable only for finding the subjective weights of sustainability indicators. There is no study which determines the combined weights including the objective and subjective weights of sustainability indicators.
- One of the effective MCDM methods, the “additive ratio assessment (ARAS)”^35^ evaluates and ranks the alternatives according to the utility function value. In this method, the ratio to the optimal value is determined to avoid the difficulties caused by different dimensions of criteria. In the context of IFSs, Mishra et al.^36–38^ proposed the ARAS-based decision support system for assessing the IT personnel selection problem from multiple criteria perspective. However, that study avoids the subjective weights of criteria, which considers the DMEs’ opinions while making a decision. In addition, the AOs used by Mishra et al.^36^ have some limitations in group decision-making process.

### Motivations and key contributions

Existing review studies contributed significantly to “sustainable development indicators (SDIs)” and its relevant subject areas; though, the existence of some important knowledge gaps motivated the present work. Some of these studies are focused upon a specific topic and some address “sustainable industrial building options (SIBOs)” as a topic amongst lots of other subtopics. Therefore, in this study, to classify the most important SDIs, a survey approach has been accomplished using literature review and specialists’ interviews. To assess the SIBOs, a hybrid MCDM technique is proposed by combining the fairly AO^39^, “MEthod based on the Removal Effects of Criteria (MEREC)”^40^, “Stepwise Weight Assessment Ratio Analysis (SWARA)”^41^ and ARAS methods with IFSs. The developed framework uses IFS theory to consider the uncertainty of information offered by the DMEs in the evaluation process.

Now, the key novelties of this work are presented as follows:

- This paper develops an innovative decision-making framework based on fairly aggregation operator, MEREC, SWARA and ARAS methods with intuitionistic fuzzy information.
- To aggregate the individual decision information and avoid the drawbacks of basic AOs of IFSs, this study proposes novel intuitionistic fuzzy fairly AOs with their desirable properties.
- In the proposed framework, an incorporated criteria weighting model is proposed by combining the MEREC model for objective weights and the SWARA model for subjective weights under intuitionistic fuzzy environment.
- This study implements the proposed decision-making framework on a case study of SIBOs assessment problem, in which the criteria weighting tool determines the priorities of the SDIs, while the present ARAS method evaluates and ranks the SIBOs under IFS context.

### Organization of this study

The remaining sections are summarized as follows: “Literature review” reviews the literature related to the sustainable industrial buildings. “New intuitionistic fuzzy fairly aggregation operators” firstly discusses the basic definitions and then proposes novel fairly AOs for intuitionistic fuzzy numbers. “New IF-MEREC-SWARA-ARAS methodology” proposes a novel decision-making framework using the MEREC, the SWARA and the ARAS approaches under IFSs setting. “Implementation of proposed method: a case study” presents a case study of SIBOs assessment from intuitionistic fuzzy perspective. In addition, this section performs the sensitivity analysis and comparative study. At last, “Conclusions” presents the concluding remarks and recommendations for future studies.

## Literature review

Here, this paper presents the comprehensive review of literature related to this study.

### Sustainable development in industrial buildings

Generally environmental aspects of sustainability for IBs are emphasized because of the high materials, energy consumption and waste generation. Despite the fact that the effects of industrial development at regional levels and on economic growth of societies are undeniable. The IB plays one of the significant roles in the SD of any society; therefore, few research efforts have been made in this direction^42–44^. Few researchers have focused their interest on ecological and economic aspects of sustainability in IBs and ignored the social aspect^9^.

Nowadays, sustainability awareness has become increasingly more significant for the society. In this respect, there are few studies that have analyzed innovation and SD together from a triple bottom line (TBL) perspective for IBs. For example, Zeng et al.^8^ utilized hierarchy cluster analysis for analyzing industrial sustainability. Chen et al.^16^ suggested an innovative framework to express the relationship among factory buildings, manufacturing equipment, the process of factory planning and TBL aspects of SD. Infante et al.^18^ considered the social, economic and environmental dimensions of sustainability in order to assess the leading corporations in the oil and gas sectors. Tan et al.^20^ gave an innovative procedure for reuse assessment of IBs under fuzzy environment.

With the use of structural equation modeling, Heravi et al.^10^ analyzed and assessed the social, environmental and economic aspects in the selection of IBs. Cuadrado et al.^17^ proposed an “analytic hierarchy process (AHP)” method for sustainability assessment of IBs. Further, Heravi et al.^11^ firstly analyzed and assessed the TBL aspects of SD in IBs. That study introduced a hybrid decision support system for evaluating IBs from sustainability perspectives. Vardopoulos^22^ proposed an application of fuzzy DEMATEL in the adaptive reuse of urban IBs. Milosevic et al.^19^ using the adjusted fuzzy AHP examined the potential for the adaptation of IBs in Nis in Serbia. Tian et al.^21^ structured an MCDM tool for evaluating their used IBs from SD perspective. Till now, no one has developed an integrated MCDM method for assessing SIBOs under IFS environment.

### MCDM methods in sustainability

MCDM methods are considered as capable tools to help the scientists and engineers in solving decision-making applications. Over the past few decades, numerous theories and methods have been presented to treat the uncertainty of human intuition. After the pioneering idea of FST^45^, several MCDM methods have been introduced within fuzzy set context^46^. After the pioneering work of Atanassov^12^, the theory of IFSs has been used more frequently to reflect the accurate semantics of DMEs.

Since its appearance, the theory of IFS and its applications have attracted more attention from scholars. For instance, Mousavi et al.^47^ introduced a novel intuitionistic fuzzy relative closeness coefficient-based model for the evaluation of construction projects. They derived the DMEs’ significance values using an innovative intuitionistic fuzzy index and criteria’ weights using the concept of closer to ideal solution and farther from negative ideal solution. Cavallaro et al.^23^ put forward a hybrid intuitionistic fuzzy MCDM model for assessing concentrated solar power technologies under IFS context. De et al.^24^ firstly constructed the credit risk evaluation index system and further, suggested a hybrid tool using the AHP with IFS. Mishra and Rani^28^ discussed a collective framework for choosing a cloud service provider from intuitionistic fuzzy perspective. Liang et al.^27^ firstly proposed some new intuitionistic fuzzy distance measures and AOs, and further applied to develop an extended “multi-attributive border approximation area comparison (MABAC)” framework for treating correlative MCDM problems. Ghaderi et al.^48^ developed a novel intuitionistic fuzzy information-based decision support system to evaluate and prioritize the decision-making units in accordance with their performances. Zhang et al.^34^ put forward a novel intuitionistic fuzzy UTASTAR model in treating the low-carbon tourism destination selection. Liu et al.^49^ recommended a latest intuitionistic fuzzy partitioned Bonferroni mean operator to treat the MCDM process. In order to assess the rooftop photovoltaic project sites, Gao et al.^26^ recommended an MCDM methodology by combining intuitionistic fuzzy score function, prospect theory, analytical network process and linear weighting technique. Ocampo et al.^29^ suggested the TOPSIS-Sort method with IFSs and demonstrated in arranging the restaurants for apparent experience of clients to COVID-19.

As the criteria weights are very important in making a decision, therefore, several weighting models have been developed in this context. To determine the objective weights of criteria, a novel MEREC model has been developed by Keshavarz-Ghorabaee et al.^40^. This method uses the removal effects of criteria in the decision matrix to derive their importance. Ecer and Aycin^50^ introduced a new decision support system using MEREC weighting-based score aggregation model and used for measuring innovation performance of G7 countries. Hezam et al.^51^ put forward an incorporated MCDM framework by combining the MEREC model with intuitionistic fuzzy double normalization-based multiple aggregation approach and applied to evaluate the alternative fuel vehicles problem. The MEREC method has combined with simple weighted sum product model for evaluate and rank the pallet trucks^52^. Recently, Keleş^53^ measured the performances through MEREC model using geometric mean and harmonic mean as multiplicative functions.

For subjective weights of criteria, Kersuliene et al.^41^ initiated the SWARA model in which the experts’ opinions are highly preferred. In comparison with AHP^54^, the SWARA approach does not involve a large number of pairwise comparisons and has high consistency. In comparison with best worst method^55^, the SWARA approach does not need to estimate multifaceted linear objective function, has minimum computational difficulty, and is effortless to utilize. In the recent times, the SWARA method has been combined with several MCDM methods under different contexts. Ghenai et al.^56^ incorporated the SWARA with ARAS method to treat the renewable energy systems (RESs) with sustainability perspectives. Further, Alipour et al.^57^ assessed the fuel cell and hydrogen components providers by means of a hybrid Pythagorean fuzzy SWARA-COPRAS approach. A hybrid Pythagorean fuzzy decision support system based on SWARA method has been developed for identifying the key barriers to the adoption of Internet of Things^58^. Yücenur and Şenol^59^ gave a novel decision-making method by combining SWARA and fuzzy “Visekriterijumska optimizacija I kompromisno resenje (VIKOR)” approaches in waste removal and formation of lean creation procedures.

In the literature, several MCDM methods have been developed to solve the real-life decision-making problems such as construction projects evaluation^47^, assessment of sustainable projects for municipality^60^, sustainable feed stocks selection and renewable products allocation^61^, brick production technologies assessment^62^ and so forth. Each method has its own advantage and disadvantage^37^. The ARAS^35^, one of the popular MCDM methods, is based on the theory that complex phenomena of the world could be exactly perceived through simple relative comparisons. In terms of SD, Esmail and Geneletti^63^ made a review of multiple criteria decision approaches in different problems of nature conservation. An integrated intuitionistic fuzzy ARAS method has suggested for assessing the multi-criteria IT personnel problem^38^. In the context of sustainability, Kandakoglu et al.^1^ gave the organized review of the work with multi-criteriain sustainability perspectives from 2010 to 2017. Rostamzadeh et al.^64^ designed an innovative fuzzy information-based ARAS methodology for “sustainable third party reverse logistics providers (S3PRLP)” assessment. Karagöz^65^ incorporated ARAS with “interval type-2 fuzzy sets (IT2FSs)” for the evaluation of recycling facility locations from SD context. Pandey et al.^66^ provide a review on decision methods under uncertainty for clean energy.

### Identification and evaluation of SDIs

**Figure 1:**
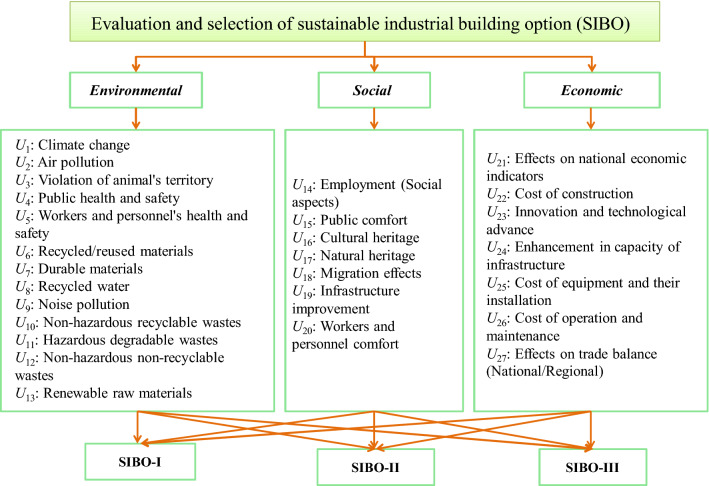
Research framework of selection sustainable industrial building options.

In the context of SD, indicators should comprise TBL perspective of sustainability. Various building assessment techniques have been presented and employed from sustainable points of view. Because of explicit characteristics and industrial activities, industrial buildings are different from residential and commercial ones. The environmental, economic and social aspects are affecting up these buildings in a diverse manner, particularly when the concern arises to the developing nations from industrial development perspective. In most of the developing countries, sustainability is not being efficiently put in practice. In this study, this paper firstly identifies the sustainability indicators based on existing studies related to IBs^10,67^. The SDIs and their references are shown in Table 1. The innovation of current research is the hybridization of SDIs and uncertain MCDM method to determine and rank SIBOs and presented in Fig. 1.

**Table 1:** Evaluation and identification of sustainable development indicators (SDIs).

Dimension	Indicators	References^a^
Environmental (EN)	*U*_1_: Climate change *U*_2_: Air pollution *U*_3_: Violation of animal's territory *U*_4_: Public health and safety *U*_5_: Workers and personnel's health and safety *U*_6_: Recycled/reused materials *U*_7_: Durable materials *U*_8_: Recycled water *U*_9_: Noise pollution *U*_10_: Non-hazardous recyclable wastes *U*_11_: Hazardous degradable wastes *U*_12_: Non-hazardous non-recyclable wastes *U*_13_: Renewable raw materials	[2], [5], [8], [9], [12], [15], [16] [3], [8], [11], [12], [16] [17], [18] [2], [3], [8], [9], [10], [11], [12], [14], [15] [2], [3], [5], [8], [9], [10], [11], [12], [14], [15], [16] [5], [8], [18] [2], [4], [7], [9], [15] [6], [18] [2], [3], [9], [10], [11], [15] [2], [3], [4], [7], [8], [9], [10], [11], [13], [15], [16] [2], [3], [4], [7], [8], [9], [10], [11], [13], [15], [16] [2], [3], [4], [7], [8], [9], [10], [11], [13], [15], [16] [3], [5], [6]
Social (SC)	*U*_14_: Employment *U*_15_: Public comfort *U*_16_: Cultural heritage *U*_17_: Natural heritage *U*_18_: Migration effects *U*_19_: Infrastructure improvement *U*_20_: Workers and personnel comfort	[3], [11], [14] [1], [2], [3], [5], [9], [10], [11], [13], [15] [3], [10], [11] [3], [7], [18] [1], [18] [11], [18] [2], [4], [7], [9], [12], [15]
Economic (EC)	*U*_21_: Effects on national economic indicators *U*_22_: Cost of construction *U*_23_: Innovation and technological advance *U*_24_: Enhancement in capacity of infrastructure *U*_25_: Cost of equipment and their installation *U*_26_: Cost of operation and maintenance *U*_27_: Effects on trade balance (National/Regional)	[17], [18] [2], [9], [15] [4], [7], [11], [12] [2], [9], [12], [15], [16] [2], [9], [15] [2], [9], [15] [1], [18]

^a^[1] Domac et al., 2005^68^; [2] Von Geibler et al., 2006^69^; [3] San-Jose et al., 2007^44^; [4] Shen et al., 2007^70^; [5] Ugwu and Haupt, 2007^71^; [6] USGBC, 2009^72^; [7] Alwaer et al., 2008^73^; [8] Aliand Al Nsairat, 2009^74^; [9] Alwaer and Clements-Croome, 2010^75^; [10] San-Jose Lombera and Garrucho Aprea, 2010^42^; [11] Shen et al., 2011^76^; [12] Bakhoum and Brown, 2012^77^; [13] Chen et al., 2012^16^; [14] Cuadrado et al., 2012^17^; [15] Larimian et al., 2013^78^; [16] Infante et al., 2013^18^; [17] Heravi et al., 2015^10^; [18] Heravi et al., 2017^11^.

## New intuitionistic fuzzy fairly aggregation operators

First of all, this section recalls the basic notions of IFSs and further introduces an innovative methodology for solving MCDM problems under IFS context.

### Preliminaries

Atanassov^12^ put forward the concept of IFSs, which is mathematically defined as

#### Definition 3.1^12^.

 An IFS $L$ on $\Theta = \left\{ {v_{1} ,\,\,v_{2} ,\,\,...,\,v_{n} } \right\}$ is given by

1 $$L = \left\{ {\left\langle {v_{i} ,\,\,b_{L} (v_{i} ),\,\,n_{L} (v_{i} )} \right\rangle \,\,:\,\,v_{i} \in \Theta } \right\},$$

wherein $b_{L} :\Theta \to [0,\,\,1]$ and $n_{L} :\Theta \to [0,\,\,1]$ presents the “belongingness degree (BD)” and “non-belongingness degree (ND)” of $v_{i}$ to $L$ in $\Theta ,$ with the constraint

2 $$0 \le b_{L} \left( {v_{i} } \right) \le 1,\quad 0 \le n_{L} \left( {v_{i} } \right) \le 1\;{\text{and}}\quad 0 \le b_{L} (v_{i} ) + n_{L} (v_{i} ) \le 1,\quad \forall \,v_{i} \in \Theta .$$

The intuitionistic index of an element $v_{i} \in \Theta$ to $L$ is

3 $$\pi_{L} \left( {v_{i} } \right) = 1 - b_{L} \left( {v_{i} } \right) - n_{L} \left( {v_{i} } \right)\quad {\text{and}}\quad 0 \le \pi_{L} \left( {v_{i} } \right) \le 1,\,\,\,\forall \,v_{i} \, \in \Theta .$$

Xu^33^ defined this term $\left\langle {b_{L} (v_{i} ),\,\,n_{L} (v_{i} )} \right\rangle$ as an “intuitionistic fuzzy number (IFN)” and denoted by $\wp = \,\left\langle {b_{\wp } ,\,n_{\wp } } \right\rangle$ which satisfies $b_{\wp } ,\,n_{\wp } \, \in \,\left[ {0,\,1} \right]$ and $0\, \le \,b_{\wp } + n_{\wp } \le \,1.$

#### Definition 3.2^79^.

 For an IFN $\wp_{j} = \left\langle {b_{j} ,\,n_{j} } \right\rangle ,$ the score and accuracy functions are defined as

4 $${\mathbb{S}}^{*} \left( {\wp_{j} } \right) = \frac{1}{2}\left( {b_{j} - n_{j} + 1} \right)$$

5 $$H\left( {\wp_{j} } \right) = \frac{1}{2}\left( {b_{j} + n_{j} } \right),$$

Clearly, $${\mathbb{S}}^{*} \left( {\wp_{j} } \right) \in \left[ {0,1} \right]$$ and $$H\left( {\wp_{j} } \right) \in \left[ {0,\,\,1} \right].$$

#### Definition 3.3^33^.

 For any two IFNs $\wp_{1} = \left\langle {b_{{\wp_{1} }} ,\,n_{{\wp_{1} }} } \right\rangle$ and $\wp_{2} = \left\langle {b_{{\wp_{2} }} ,\,n_{{\wp_{2} }} } \right\rangle ,$ and $\alpha \, > \,0.$ Then, the basic operations on IFNs are described as.

(i) $$\wp_{1}^{c} = \left\langle {n_{{\wp_{1} }} ,\,b_{{\wp_{1} }} } \right\rangle ,\,\,\wp_{2}^{c} = \left\langle {n_{{\wp_{2} }} ,\,b_{{\wp_{2} }} } \right\rangle ;$$
(ii) $$\wp_{1} \oplus \,\wp_{2} \, = \left\langle {b_{{\wp_{1} }} + \,b_{{\wp_{2} }} - \,b_{{\wp_{1} }} \,b_{{\wp_{2} }} ,\,n_{{\wp_{1} }} \,n_{{\wp_{2} }} } \right\rangle ;$$
(iii) $$\wp_{1} \otimes \,\wp_{2} \, = \left\langle {b_{{\wp_{1} }} \,b_{{\wp_{2} }} ,\,n_{{\wp_{1} }} + \,n_{{\wp_{2} }} - \,n_{{\wp_{1} }} \,n_{{\wp_{2} }} } \right\rangle ;$$
(iv) $$\wp_{1} \cap \,\wp_{2} \, = \left\langle {\min \left\{ {b_{{\wp_{1} }} ,\,b_{{\wp_{2} }} } \right\},\,\max \left\{ {n_{{\wp_{1} }} ,\,n_{{\wp_{2} }} } \right\}} \right\rangle ;$$
(v) $$\wp_{1} \cup \,\wp_{2} \, = \left\langle {\max \left\{ {b_{{\wp_{1} }} ,\,b_{{\wp_{2} }} } \right\},\,\min \left\{ {n_{{\wp_{1} }} ,\,n_{{\wp_{2} }} } \right\}} \right\rangle ;$$
(vi) $$\alpha \,\wp_{1} \, = \left\langle {\left( {1 - \left( {1 - b_{{\wp_{1} }} } \right)^{\alpha } } \right),\,n_{{\wp_{1} }}^{\alpha } } \right\rangle ;$$
(vii) $$\wp_{1}^{\alpha } \, = \left\langle {b_{{\wp_{1} }}^{\alpha } ,\,\left( {1 - \left( {1 - \,n_{{\wp_{1} }} } \right)^{\alpha } } \right)} \right\rangle .$$

If $$b_{{\wp_{1} }} \, = \,n_{{\wp_{1} }}$$ and $$b_{{\wp_{2} }} \, = \,n_{{\wp_{2} }} ,$$ then by Definition 3.3, we obtain $$b_{{\wp_{1} \oplus \wp_{2} }} \, \ne \,n_{{\wp_{1} \oplus \wp_{2} }} ,$$$$b_{{\wp_{1} \otimes \wp_{2} }} \, \ne \,n_{{\wp_{1} \otimes \wp_{2} }} ,$$$$b_{{\alpha \,\wp_{1} }} \, \ne \,n_{{\alpha \,\wp_{1} }}$$ and $$b_{{\wp_{1}^{\alpha } }} \, \ne \,n_{{\wp_{1}^{\alpha } }} .$$ For example, if $$\wp_{1} = \left\langle {0.3,\,0.3} \right\rangle$$ and $$\wp_{2} = \left\langle {0.4,\,0.4} \right\rangle ,$$ then the basic operations given in Definition 3.3 are as follows:

(i) $$\wp_{1}^{c} = \left\langle {0.3,\,0.3} \right\rangle ,\,\,\wp_{2}^{c} = \left\langle {0.4,\,0.4} \right\rangle ;$$
(ii) $\wp_{1} \oplus \,\wp_{2} \, = \left\langle {0.58,\,0.12} \right\rangle ,$ where $b_{{\wp_{1} \oplus \wp_{2} }} \, = \,0.58$ and $n_{{\wp_{1} \oplus \wp_{2} }} \, = \,0.12,$ therefore,$b_{{\wp_{1} \oplus \wp_{2} }} \ne \,n_{{\wp_{1} \oplus \wp_{2} }} ;$
(iii) $\wp_{1} \otimes \wp_{2} \, = \left\langle {0.12,\,0.58} \right\rangle ,$ where $b_{{\wp_{1} \otimes \wp_{2} }} \, = \,0.12$ and $n_{{\wp_{1} \otimes \wp_{2} }} \, = \,0.58,$ therefore, $b_{{\wp_{1} \otimes \wp_{2} }} \ne \,n_{{\wp_{1} \otimes \wp_{2} }} ;$
(iv) $$\wp_{1} \cap \,\wp_{2} \, = \left\langle {0.3,\,0.4} \right\rangle ;$$
(v) $$\wp_{1} \cup \,\wp_{2} \, = \left\langle {0.4,\,0.3} \right\rangle ;$$
(vi) $\alpha \,\wp_{1} \, = \left\langle {\left( {1 - \left( {1 - \,0.3} \right)^{\alpha } } \right),\,0.3^{\alpha } } \right\rangle \, = \,\left\langle {0.1633,\,0.5477} \right\rangle$ for $\alpha \, = \,0.5,$ where $b_{{\alpha \,\wp_{1} }} \, = \,0.1633$ and $n_{{\alpha \,\wp_{1} }} \, = \,0.5477,$ therefore, $b_{{\alpha \,\wp_{1} }} \, \ne \,n_{{\alpha \,\wp_{1} }} ;$
(vii) $\wp_{1}^{\alpha } \, = \left\langle {0.3^{\alpha } ,\,\left( {1 - \left( {1 - \,0.3} \right)^{\alpha } } \right)} \right\rangle \, = \,\left\langle {0.5477,\,0.1633} \right\rangle$ for $\alpha \, = \,0.5,$ where $b_{{\wp_{1}^{\alpha } }} \, = \,0.5477$ and $n_{{\wp_{1}^{\alpha } }} \, = \,0.1633,$ therefore, $b_{{\wp_{1}^{\alpha } }} \, \ne \,n_{{\wp_{1}^{\alpha } }} .$

Here, none of the operations $$\wp_{1} \oplus \,\wp_{2} ,$$$$\wp_{1} \otimes \,\wp_{2} ,$$$$\alpha \,\wp_{1} ,$$$$\wp_{1}^{\alpha }$$ found to be fair or neutral in reality. To handle this issue, this paper introduces fairly operations on IFNs in next subsection.

### Fairly operations on IFNs

In this part of the study, firstly fairly operations on IFNs are defined and further discussed their properties.

#### Definition 3.4^39^.

 For any two IFNs $\wp_{1} \, = \,\left\langle {b_{{\wp_{1} }} ,\,n_{{\wp_{1} }} } \right\rangle$ and $\wp_{2} \, = \,\left\langle {b_{{\wp_{2} }} ,\,n_{{\wp_{2} }} } \right\rangle ,$ and $\alpha \, > \,0.$ The fairly operations are defined on IFNs, which as

(i) $$\wp_{1} \,\tilde{ \otimes }\,\wp_{2} \, = \,\left\langle {\left( {\left( {\frac{{b_{{\wp_{1} }} \,b_{{\wp_{2} }} }}{{b_{{\wp_{1} }} \,b_{{\wp_{2} }} + \,n_{{\wp_{1} }} \,n_{{\wp_{2} }} }}} \right)\, \times \left( {1 - \,\left( {1 - b_{{\wp_{1} }} - \,n_{{\wp_{1} }} } \right)\,\left( {1 - b_{{\wp_{2} }} - \,n_{{\wp_{2} }} } \right)} \right)} \right),} \right.$$
  $$\left. {\left( {\left( {\frac{{n_{{\wp_{1} }} \,n_{{\wp_{2} }} }}{{b_{{\wp_{1} }} \,b_{{\wp_{2} }} + \,n_{{\wp_{1} }} \,n_{{\wp_{2} }} }}} \right)\, \times \left( {1 - \,\left( {1 - b_{{\wp_{1} }} - \,n_{{\wp_{1} }} } \right)\,\left( {1 - b_{{\wp_{2} }} - \,n_{{\wp_{2} }} } \right)} \right)} \right)} \right\rangle ;$$
(ii) $$\alpha * \,\wp_{1} \, = \,\left\langle {\left( {\left( {\frac{{b_{{\wp_{1} }}^{\alpha } }}{{b_{{\wp_{1} }}^{\alpha } \, + \,n_{{\wp_{1} }}^{\alpha } }}} \right)\, \times \,\left( {1\, - \,\left( {1 - \,b_{{\wp_{1} }} - \,n_{{\wp_{1} }} } \right)^{\alpha } } \right)} \right),\,\left( {\left( {\frac{{n_{{\wp_{1} }}^{\alpha } }}{{b_{{\wp_{1} }}^{\alpha } \, + \,n_{{\wp_{1} }}^{\alpha } }}} \right)\, \times \,\left( {1\, - \,\left( {1 - \,b_{{\wp_{1} }} - \,n_{{\wp_{1} }} } \right)^{\alpha } } \right)} \right)} \right\rangle .$$

#### Proposition 3.1

*Let us consider that* $\wp_{1} \, = \,\left\langle {b_{{\wp_{1} }} ,\,n_{{\wp_{1} }} } \right\rangle$ *and* $\wp_{2} \, = \,\left\langle {b_{{\wp_{2} }} ,\,n_{{\wp_{2} }} } \right\rangle$ *be two IFNs and* $\alpha \, > \,0.$ *If* $b_{{\wp_{1} }} \, = \,n_{{\wp_{1} }}$ *and* $b_{{\wp_{2} }} \, = \,n_{{\wp_{2} }} ,$ *then*

(i) $$b_{{\wp_{1} \tilde{ \otimes }\wp_{2} }} \, = \,n_{{\wp_{1} \tilde{ \otimes }\wp_{2} }} ,$$
(ii) $$b_{{\alpha \, * \wp_{1} }} \, = \,n_{{\alpha * \,\wp_{1} }} .$$

#### Proof

(i) Since $b_{{\wp_{1} }} \, = \,n_{{\wp_{1} }}$ and $b_{{\wp_{2} }} \, = \,n_{{\wp_{2} }} ,$ then
  $$\frac{{b_{{\wp_{1} \tilde{ \otimes }\wp_{2} }} }}{{n_{{\wp_{1} \tilde{ \otimes }\wp_{2} }} }}\, = \,\frac{{\left( {\frac{{b_{{\wp_{1} }} \,b_{{\wp_{2} }} }}{{b_{{\wp_{1} }} \,b_{{\wp_{2} }} + \,n_{{\wp_{1} }} \,n_{{\wp_{2} }} }}} \right)\, \times \left( {1 - \,\left( {1 - b_{{\wp_{1} }} - \,n_{{\wp_{1} }} } \right)\,\left( {1 - b_{{\wp_{2} }} - \,n_{{\wp_{2} }} } \right)} \right)}}{{\left( {\frac{{n_{{\wp_{1} }} \,n_{{\wp_{2} }} }}{{b_{{\wp_{1} }} \,b_{{\wp_{2} }} + \,n_{{\wp_{1} }} \,n_{{\wp_{2} }} }}} \right)\, \times \left( {1 - \,\left( {1 - b_{{\wp_{1} }} - \,n_{{\wp_{1} }} } \right)\,\left( {1 - b_{{\wp_{2} }} - \,n_{{\wp_{2} }} } \right)} \right)}}\, = \,\left( {\frac{{b_{{\wp_{1} }} \,b_{{\wp_{2} }} }}{{n_{{\wp_{1} }} \,n_{{\wp_{2} }} }}} \right)\, = 1.$$

Thus, $$b_{{\wp_{1} \tilde{ \otimes }\wp_{2} }} \, = \,n_{{\wp_{1} \tilde{ \otimes }\wp_{2} }} .$$

(b) By following (i), we can show that $b_{{\alpha \, * \wp_{1} }} \, = \,n_{{\alpha * \,\wp_{1} }}$ for $b_{{\wp_{1} }} \, = \,n_{{\wp_{1} }}$ and $b_{{\wp_{2} }} \, = \,n_{{\wp_{2} }} .$

For the same example as given in section "Intuitionistic fuzzy weighted fairly AO", we get $\wp_{1} \,\tilde{ \otimes }\,\wp_{2} \, = \left\langle {0.46,\,0.46} \right\rangle ,$ where $b_{{\wp_{1} \,\tilde{ \otimes }\,\wp_{2} \,}} = \,n_{{\wp_{1} \,\tilde{ \otimes }\,\wp_{2} \,}} .$ Also, we obtain $\alpha * \,\wp_{1} \, = \,\left\langle {0.1838,\,0.1838} \right\rangle ,$ where $b_{{\alpha * \,\wp_{1} }} \, = \,n_{{\alpha * \,\wp_{1} }} .$

#### Theorem 3.1

*For any two IFNs* $\wp_{1} \, = \,\left\langle {b_{{\wp_{1} }} ,\,n_{{\wp_{1} }} } \right\rangle$ *and* $\wp_{2} \, = \,\left\langle {b_{{\wp_{2} }} ,\,n_{{\wp_{2} }} } \right\rangle ,$ *and three real numbers* $\alpha ,\,\alpha_{1} ,\,\alpha_{2} \, > \,0,$ *we obtain*

(i) $$\wp_{1} \,\tilde{ \otimes }\,\wp_{2} \, = \,\wp_{2} \,\tilde{ \otimes }\,\wp_{1} ,$$
(ii) $$\alpha * \left( {\wp_{1} \,\tilde{ \otimes }\,\wp_{2} } \right)\, = \,\left( {\alpha * \wp_{1} } \right)\tilde{ \otimes }\left( {\alpha * \wp_{2} } \right),$$
(iii) $$\left( {\alpha_{1} + \,\alpha_{2} } \right) * \,\wp_{1} \, = \,\left( {\alpha_{1} * \wp_{1} } \right)\tilde{ \otimes }\left( {\alpha_{2} * \wp_{1} } \right).$$

#### Proof

It is easy to prove by Definition 3.4, thus, the proof is omitted.

### Intuitionistic fuzzy weighted fairly AO

In this section, weighted fairly AO is developed for IFNs. Further, their properties are presented in details.

#### Definition 3.6.

Let $\wp_{j} \, = \,\left\langle {b_{j} ,\,n_{j} } \right\rangle ;\,\,j\, = 1(1)t$ be the set of IFNs. Then the “intuitionistic fuzzy weighted fairly AO (IFWFAO)” is given by

$$IFWFAO\left( {\wp_{1} ,\,\wp_{2} ,\,...,\,\wp_{t} } \right) = \,\left( {\omega_{1} * \,\wp_{1} } \right)\,\tilde{ \otimes }\,\left( {\omega_{2} * \,\wp_{2} } \right)\,\tilde{ \otimes }\,\left( {\omega_{3} \, * \wp_{3} } \right)\,\tilde{ \otimes }...\tilde{ \otimes }\left( {\omega_{t} * \,\wp_{t} } \right),$$

where $\omega_{j}$ is the weight of $\wp_{j} \,\left( {j\, = 1\left( 1 \right)t} \right)$ satisfying $\omega_{j} \, > \,0$ and $\sum\limits_{j\, = 1}^{t} {\omega_{j} } = 1.$

#### Theorem 3.2

*The aggregated value by using IFWFAO is also an IFN and given by*

6 $$\begin{gathered} IFWFAO(\wp_{1} ,\,\wp_{2} ,\,...,\,\wp_{t} ) = \,\left( \begin{gathered} \left( {\tfrac{{\prod\limits_{j = 1}^{t} {\left( {b_{j} } \right)^{{\omega_{j} }} } }}{{\prod\limits_{j = 1}^{t} {\left( {b_{j} } \right)^{{\omega_{j} }} } + \,\prod\limits_{j = 1}^{t} {\left( {n_{j} } \right)^{{\omega_{j} }} } }} \times \left( {1 - \prod\limits_{j = 1}^{t} {\left( {1 - \,b_{j} - n_{j} } \right)^{{\omega_{j} }} } } \right)} \right), \hfill \\ \left( {\tfrac{{\prod\limits_{j = 1}^{t} {\left( {n_{j} } \right)^{{\omega_{j} }} } }}{{\prod\limits_{j = 1}^{t} {\left( {b_{j} } \right)^{{w_{j} }} } + \,\prod\limits_{j = 1}^{t} {\left( {n_{j} } \right)^{{w_{j} }} } }} \times \left( {1 - \prod\limits_{j = 1}^{t} {\left( {1 - \,b_{j} - n_{j} } \right)^{{\omega_{j} }} } } \right)} \right) \hfill \\ \end{gathered} \right), \hfill \\ \,\,\,\,\,\,\,\,\,\,\,\,\,\,\,\,\,\,\,\,\,\,\,\,\,\,\,\,\,\,\,\,\,\,\,\,\,\,\,\,\,\,\,\,\,\,\,\,\,\, \hfill \\ \end{gathered}$$

where $\omega_{j}$ is the weight of $\wp_{j} \,\left( {j\, = 1\left( 1 \right)t} \right)$ satisfying $\omega_{j} \, > \,0$ and $\sum\limits_{j\, = 1}^{t} {\omega_{j} } = 1.$

#### Proof

In the following, we will prove Eq. (6) with the use of mathematical induction. It is evident that Eq. (6) is true for $t = \,1.$ Suppose that Eq. (6) is true for $t\, = \,k,$ therefore

$$\begin{gathered} IFWFAO(\wp_{1} ,\,\wp_{2} ,\,...,\,\wp_{m} ) = \,\left\langle \begin{gathered} \left( {\tfrac{{\prod\limits_{j = 1}^{m} {\left( {b_{j} } \right)^{{\omega_{j} }} } }}{{\prod\limits_{j = 1}^{m} {\left( {b_{j} } \right)^{{\omega_{j} }} } + \,\prod\limits_{j = 1}^{m} {\left( {n_{j} } \right)^{{\omega_{j} }} } }} \times \left( {1 - \prod\limits_{j = 1}^{m} {\left( {1 - \,b_{j} - n_{j} } \right)^{{\omega_{j} }} } } \right)} \right), \hfill \\ \left( {\tfrac{{\prod\limits_{j = 1}^{m} {\left( {n_{j} } \right)^{{\omega_{j} }} } }}{{\prod\limits_{j = 1}^{m} {\left( {b_{j} } \right)^{{w_{j} }} } + \,\prod\limits_{j = 1}^{m} {\left( {n_{j} } \right)^{{w_{j} }} } }} \times \left( {1 - \prod\limits_{j = 1}^{m} {\left( {1 - \,b_{j} - n_{j} } \right)^{{\omega_{j} }} } } \right)} \right) \hfill \\ \end{gathered} \right\rangle . \hfill \\ \,\,\,\,\,\,\,\,\,\,\,\,\,\,\,\,\,\,\,\,\,\,\,\,\,\,\,\,\,\,\,\,\,\,\,\,\,\,\,\,\,\,\,\,\,\,\,\,\,\, \hfill \\ \end{gathered}$$

When $t\, = \,m + 1,$ we have

$$IFWFAO(\wp_{1} ,\,\wp_{2} ,\,...,\,\wp_{m} ,\,\wp_{m + 1} ) = \,\left( {\omega_{1} * \,\wp_{1} } \right)\,\tilde{ \otimes }\,\left( {\omega_{2} * \,\wp_{2} } \right)\,\tilde{ \otimes }\,\left( {\omega_{3} \, * \wp_{3} } \right)\,\tilde{ \otimes }...\tilde{ \otimes }\left( {\omega_{m} * \,\wp_{m} } \right)\tilde{ \otimes }\left( {\omega_{m + 1} * \,\wp_{m + 1} } \right)$$

$$= \left\langle \begin{gathered} \left( {\tfrac{{\prod\limits_{j = 1}^{m} {\left( {b_{j} } \right)^{{\omega_{j} }} } }}{{\prod\limits_{j = 1}^{m} {\left( {b_{j} } \right)^{{\omega_{j} }} } + \,\prod\limits_{j = 1}^{m} {\left( {n_{j} } \right)^{{\omega_{j} }} } }} \times \left( {1 - \prod\limits_{j = 1}^{m} {\left( {1 - \,b_{j} - n_{j} } \right)^{{\omega_{j} }} } } \right)} \right), \hfill \\ \left( {\tfrac{{\prod\limits_{j = 1}^{m} {\left( {n_{j} } \right)^{{\omega_{j} }} } }}{{\prod\limits_{j = 1}^{m} {\left( {b_{j} } \right)^{{w_{j} }} } + \,\prod\limits_{j = 1}^{m} {\left( {n_{j} } \right)^{{w_{j} }} } }} \times \left( {1 - \prod\limits_{j = 1}^{m} {\left( {1 - \,b_{j} - n_{j} } \right)^{{\omega_{j} }} } } \right)} \right) \hfill \\ \end{gathered} \right\rangle \tilde{ \otimes }\left\langle \begin{gathered} \left( {\tfrac{{\left( {b_{m + 1} } \right)^{{\omega_{m + 1} }} }}{{\left( {b_{m + 1} } \right)^{{\omega_{m + 1} }} + \,\left( {n_{m + 1} } \right)^{{\omega_{m + 1} }} }} \times \left( {1 - \left( {1 - \,b_{m + 1} - n_{m + 1} } \right)^{{\omega_{m + 1} }} } \right)} \right), \hfill \\ \left( {\tfrac{{\left( {n_{m + 1} } \right)^{{\omega_{m + 1} }} }}{{\left( {b_{m + 1} } \right)^{{\omega_{m + 1} }} + \,\left( {n_{m + 1} } \right)^{{\omega_{m + 1} }} }} \times \left( {1 - \left( {1 - \,b_{m + 1} - n_{m + 1} } \right)^{{\omega_{m + 1} }} } \right)} \right) \hfill \\ \end{gathered} \right\rangle$$

Thus, by Definition 3.4, we have

$$\begin{gathered} IFWFAO(\wp_{1} ,\,\wp_{2} ,\,...,\,\wp_{m} ,\,\wp_{m + 1} ) = \,\left\langle \begin{gathered} \left( {\tfrac{{\prod\limits_{j = 1}^{m + 1} {\left( {b_{j} } \right)^{{\omega_{j} }} } }}{{\prod\limits_{j = 1}^{m + 1} {\left( {b_{j} } \right)^{{\omega_{j} }} } + \,\prod\limits_{j = 1}^{m + 1} {\left( {n_{j} } \right)^{{\omega_{j} }} } }} \times \left( {1 - \prod\limits_{j = 1}^{m + 1} {\left( {1 - \,b_{j} - n_{j} } \right)^{{\omega_{j} }} } } \right)} \right), \hfill \\ \left( {\tfrac{{\prod\limits_{j = 1}^{m + 1} {\left( {n_{j} } \right)^{{\omega_{j} }} } }}{{\prod\limits_{j = 1}^{m + 1} {\left( {b_{j} } \right)^{{w_{j} }} } + \,\prod\limits_{j = 1}^{m + 1} {\left( {n_{j} } \right)^{{w_{j} }} } }} \times \left( {1 - \prod\limits_{j = 1}^{m + 1} {\left( {1 - \,b_{j} - n_{j} } \right)^{{\omega_{j} }} } } \right)} \right) \hfill \\ \end{gathered} \right\rangle . \hfill \\ \,\,\,\,\,\,\,\,\,\,\,\,\,\,\,\,\,\,\,\,\,\,\,\,\,\,\,\,\,\,\,\,\,\,\,\,\,\,\,\,\,\,\,\,\,\,\,\,\,\, \hfill \\ \end{gathered}$$

i.e., Eq. (6) holds for $t\, = \,m + 1.$

Therefore, Eq. (6) holds for all *t*. This completes the proof.

#### Definition 3.6

Consider a collection of IFNs $\wp_{j} \, = \,\left( {b_{j} ,\,n_{j} } \right)\,(j\, = \,1(1)t).$ Let $\omega \, = \,\left( {\omega_{1} ,\,\omega_{2} ,\,...,\,\omega_{t} } \right)^{T}$ be the weight vector of $\wp_{j} \,(j\, = 1(1)\,n),$ satisfying that $\omega_{j} \, > \,0$ and $\sum\limits_{j\, = 1}^{t} {\omega_{j} } = 1.$ Then the “intuitionistic fuzzy ordered weighted fairly AO (IFOWFAO)” is defined as

$$IFOWFAO\left( {\wp_{1} ,\,\wp_{2} ,\,...,\,\wp_{t} } \right) = \,\left( {\omega_{1} * \,\wp_{\sigma (1)} } \right)\,\tilde{ \otimes }\,\left( {\omega_{2} * \,\wp_{\sigma (2)} } \right)\,\tilde{ \otimes }\,\left( {\omega_{3} \, * \wp_{\sigma (3)} } \right)\,\tilde{ \otimes }...\tilde{ \otimes }\left( {\omega_{t} * \,\wp_{\sigma (t)} } \right),$$

wherein $\left( {\sigma (1),\,\sigma (2),\,...,\,\sigma (t)} \right)$ denotes the permutation of $\left( {1,\,2,\,...,\,t} \right)$ with $\wp_{\sigma (j - 1)} \, \ge \,\wp_{\sigma (j)} ,\,\,\forall \,j = \,2,\,3,\,...,\,t.$

#### Theorem 3.3

*The aggregated value by using IFOWFAO is also an IFN, defined by*

7 $$IFOWFAO(\wp_{1} ,\,\wp_{2} ,\,...,\,\wp_{t} ) = \,\left\langle \begin{gathered} \left( {\tfrac{{\prod\limits_{j = 1}^{t} {\left( {b_{\sigma (j)} } \right)^{{\omega_{j} }} } }}{{\prod\limits_{j = 1}^{t} {\left( {b_{\sigma (j)} } \right)^{{\omega_{j} }} } + \,\prod\limits_{j = 1}^{t} {\left( {n_{\sigma (j)} } \right)^{{\omega_{j} }} } }} \times \left( {1 - \prod\limits_{j = 1}^{t} {\left( {1 - \,b_{\sigma (j)} - n_{\sigma (j)} } \right)^{{\omega_{j} }} } } \right)} \right), \hfill \\ \left( {\tfrac{{\prod\limits_{j = 1}^{t} {\left( {n_{\sigma (j)} } \right)^{{\omega_{j} }} } }}{{\prod\limits_{j = 1}^{t} {\left( {b_{\sigma (j)} } \right)^{{w_{j} }} } + \,\prod\limits_{j = 1}^{t} {\left( {n_{\sigma (j)} } \right)^{{w_{j} }} } }} \times \left( {1 - \prod\limits_{j = 1}^{t} {\left( {1 - \,b_{\sigma (j)} - n_{\sigma (j)} } \right)^{{\omega_{j} }} } } \right)} \right) \hfill \\ \end{gathered} \right\rangle .$$

#### Proof

Similar as Theorem 3.2.

Based on Theorems 3.2 and 3.3, we derive the following properties:

#### Property 3.1 (Idempotency).

 If all IFNs $\wp_{j} = \,\left( {b_{j} ,\,n_{j} } \right)\,(j\, = 1(1)t)$ are equal, i.e., $\wp_{j} = \,\wp \, = \,\left( {b,\,n} \right),$ then $IFWFAO\left( {\wp_{1} ,\,\wp_{2} ,\,...,\,\wp_{t} } \right) = \,\wp$ and $IFOWFAO\left( {\wp_{1} ,\,\wp_{2} ,\,...,\,\wp_{t} } \right) = \,\wp .$

#### Property 3.2 (Boundedness).

 For a collection of IFNs $\wp_{j} = \,\left( {b_{j} ,\,n_{j} } \right)\,(j\, = 1(1)t),$ let $\wp^{ - } = \,\mathop {\min }\limits_{j} \,\wp_{j}$ and $\wp^{ + } = \,\mathop {\max }\limits_{j} \,\wp_{j} .$ Then, we have $\wp^{ - } \le \,IFWFAO\left( {\wp_{1} ,\,\wp_{2} ,\,...,\,\wp_{t} } \right)\, \le \,\wp^{ + }$ and $\wp^{ - } \le \,IFOWFAO\left( {\wp_{1} ,\,\wp_{2} ,\,...,\,\wp_{t} } \right)\, \le \,\wp^{ + } .$

#### Property 3.3 (Monotonicity).

 Let $\wp_{j} \, = \,\left( {b_{{\wp_{j} }} ,\,n_{{\wp_{j} }} } \right)$ and $\Im_{j} \, = \,\left( {b_{{\Im_{j} }} ,\,n_{{\Im_{j} }} } \right)(j\, = 1(1)t)$ be the group of FFNs and $\wp_{j} \, \le \,\Im_{j} ,$ i.e., $b_{{\wp_{j} }} \, \le \,b_{{\Im_{j} }}$ and $n_{{\wp_{j} }} \, \ge \,n_{{\Im_{j} }} ,\,\forall \,\,j\, = 1(1)t.$ Then

$$IFWFAO\left( {\wp_{1} ,\,\wp_{2} ,\,...,\,\wp_{t} } \right)\, \le \,IFWFAO\left( {\Im_{1} ,\,\Im_{2} ,\,...,\,\Im_{t} } \right)$$

and

$$IFOWFAO\left( {\wp_{1} ,\,\wp_{2} ,\,...,\,\wp_{t} } \right)\, \le \,IFOWFAO\left( {\Im_{1} ,\,\Im_{2} ,\,...,\,\Im_{t} } \right).$$

## New IF-MEREC-SWARA-ARAS methodology

**Figure 2:**
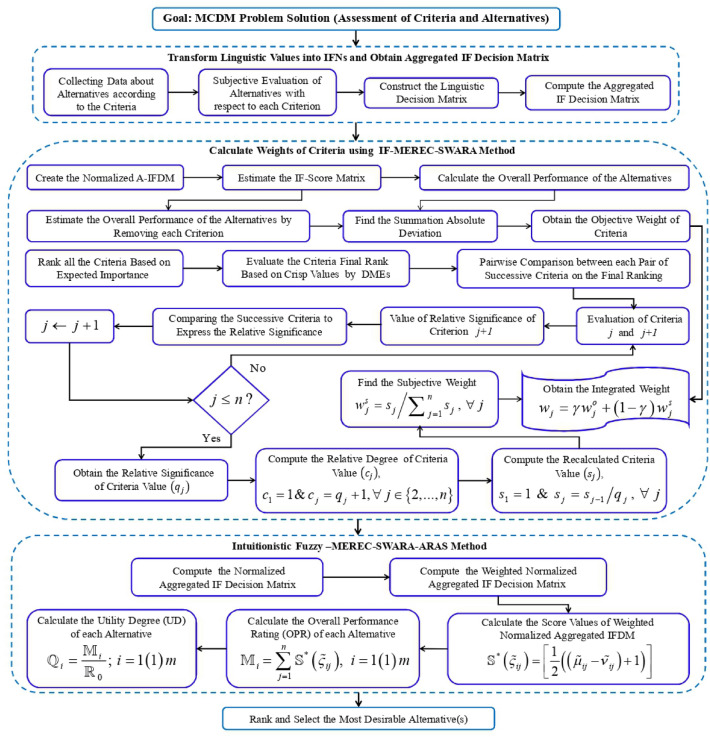
Diagrammatic representation of IF-MEREC-SWARA-ARAS method.

In this part of the study, a decision support system is established to prioritize the list of alternatives based on multiple criteria on IFSs setting. In this respect, an integrated IF-MEREC-SWARA-ARAS technique is given for solving MCDM issues with fully unknown criteria and DMEs’ weights. In this process, the IF-MEREC-SWARA model has employed for deriving the combined weights of indicators. In addition, the IFWFA operator is utilized for aggregating the single DME’s opinions. On the other hand, the ARAS technique has employed for estimating the prioritization of the alternatives. The outline of the IF-MEREC-SWARA-ARAS methodology is depicted as follows (see Fig. 2):

Step 1: Create the “linguistic decision matrix (LDM)”.

A team of DMEs $T = \left\{ {t_{1} ,\,t_{2} ,...,\,t_{\ell } } \right\}$ has been made to find the best option(s) among a set of alternatives/options $M = \left\{ {M_{1} ,\,M_{2} ,...,\,M_{m} } \right\}$ over the criterion set $U = \left\{ {U_{1} ,\,U_{2} ,...,U_{n} } \right\}.$ Consider that $Z^{\left( k \right)} = \left( {\xi_{ij}^{\left( k \right)} } \right)_{m\, \times \,n}$ be a “linguistic decision-matrix (LDM)” offered by the DMEs, wherein $\xi_{ij}^{(k)}$ refers to the performance of *M*_*i*_ over a criterion $U_{j}$ in terms of “linguistic values (LVs)” given by $k{\text{th}}$ DME.

Step 2: Evaluation of DMEs’ weights.

Let $\eta = \left( {\eta_{1} ,\,\eta_{2} ,...,\,\eta_{\ell } } \right)^{T}$ be a set of DMEs’ weights. Let $R_{k} = \left( {b_{k} ,\,n_{k} } \right)$ be an IFN for the evaluation of *k*th DME. Then, the weighting formula for *k*^th^ expert is shown in Eq. (8).

8 $$\eta_{k} = \frac{{\left( {b_{k} + \pi_{k} \left( {\frac{{b_{k} }}{{b_{k} + n_{k} }}} \right)} \right)}}{{\sum\limits_{k = 1}^{\ell } {\left( {b_{k} + \pi_{k} \left( {\frac{{b_{k} }}{{b_{k} + n_{k} }}} \right)} \right)} }},\,\,\,\,\,k = 1\left( 1 \right)\ell .$$

Step 3: Build the “aggregated intuitionistic fuzzy decision-matrix (A-IFDM)”.

To determine the A-IFDM, it is essential to merge each individual LDM into an A-IFDM in accordance with DMEs’ opinions. In this regard, IFWFAO is applied and then obtained the A-IFDM ${\mathbb{R}} = \left( {\overline{\xi }_{ij} } \right)_{m\, \times \,n} ,$ where

9 $$\overline{\xi }_{ij} = IFWA_{\eta } \,\left( {\xi_{ij}^{(1)} ,\xi_{ij}^{(2)} ,...,\xi_{ij}^{(\ell )} } \right) = \,\left( {1 - \prod\limits_{k = \,1}^{\ell } {\left( {1 - \,b_{k} } \right)^{{\eta_{k} }} } \,,\,\prod\limits_{k = 1}^{\ell } {\left( {n_{k} } \right)^{{\eta_{k} }} } } \right).$$

Step 4: Combined weight-determining model for criteria weights.

In this step, firstly the objective and subjective weights of criteria are derived, and then integrated to find the final criteria weights. This process involves the following cases:

Case I: IF-MEREC for objective weights of criteria^80^.

This model has the following steps:

Step 4a: Compute the “normalized A-IFDM (NA-IFDM)”.

In the process of MCDM, the A-IFDM ${\mathbb{R}} = \left( {\overline{\xi }_{ij} } \right)_{m\, \times \,n}$ is transformed into NA-IFDM ${\mathbb{N}} = \left( {\varsigma_{ij} } \right)_{m\, \times \,n}$ such that

10 $$\varsigma_{ij} = \left\{ \begin{gathered} \overline{\xi }_{ij} = \left( {\overline{b}_{ij} ,\overline{n}_{ij} } \right),\,\,\,\,\,\,\,\,\,\,\,\,\,\,\,\,j \in U_{b} \hfill \\ \left( {\overline{\xi }_{ij} } \right)^{c} = \left( {\overline{n}_{ij} ,\overline{b}_{ij} } \right),\,\,\,\,\,\,\,\,\,j \in U_{c} \hfill \\ \end{gathered} \right.,$$

where *U*_*b*_ and *U*_*n*_ represent the benefit and cost types of criteria sets, respectively.

Step 4b: Compute the score matrix.

Using Eq. (11), find the score matrix $\Omega = \left( {\eta_{ij} } \right)_{m\, \times \,n}$ of each IFN $\varsigma_{ij} .$

11 $$\eta_{ij} = \frac{1}{2}\left( {\left( {\overline{b}_{ij} } \right) - \left( {\overline{n}_{ij} } \right) + 1} \right).$$

Step 4c: Determine the overall performance of the options.

Based on step 4b, we can ensure that the smaller values of $\eta_{ij}$ provides the better values of the performances. To compute the overall performance of each option, Eq. (12) is utilized.

12 $$S_{i} = \,ln\left( {1 + \,\left( {\frac{1}{n}\,\sum\limits_{j} {\left| {ln\left( {\eta_{ij} } \right)} \right|} } \right)} \right).$$

Step 4d: Find the performance of the options by removal of criteria separately.

The performance of each option is determined by removing each criterion individually, given by

13 $$S_{ij}^{^{\prime}} = ln\left( {1 + \left( {\frac{1}{n}\sum\limits_{k,k \ne j} {\left| {ln\left( {\eta_{ik} } \right)} \right|} } \right)} \right).$$

Thus, *n* sets of performances are achieved concerning *n* criteria.

Step 4e: Sum of absolute deviations.

Let $V_{j}$ denote the removal effect of *j*th criterion, which is calculated as

14 $$V_{j} = \sum\limits_{i} {\left| {S_{ij}^{^{\prime}} - S_{i} } \right|} .$$

Step 4f: Derive the objective weights of criteria.

The objective weight of each criterion is determined by Eq. (15).

15 $$w_{j}^{o} = \frac{{V_{j} }}{{\sum\limits_{j = 1}^{n} {V_{j} } }}.$$

Case II: IF-SWARA approach for subjective weights of criteria.

The SWARA approach starts by prioritizing the attributes and then pairwise assesses the higher rank attribute to the lower rank attribute. The procedures are as follows:

Step 4g: Compute the score values using Definition 3.2.

Step 4h: Based on DMEs’ preferences, grade the criteria from most significance to the least significance.

Step 4i: The relative significance is derived from the attributes that are placed in the second spot, and succeeding relative significance is found with the attribute $j$ and attribute $j - 1.$

Step 4j: The relative degree $c_{j}$ is assessed as follows:

16 $$c_{j} = \left\{ \begin{gathered} 1,\,\,\,\,\,\,\,\,\,\,\,j = 1 \hfill \\ q_{j} + 1,\,\,j > 1, \hfill \\ \end{gathered} \right.$$

wherein $q_{j}$ is the relative significance of average degree.

Step 4k: The initial weight $s_{j}$ is obtained using

17 $$s_{j} = \left\{ \begin{gathered} 1,\,\,\,\,\,\,\,\,\,j = 1 \hfill \\ \frac{{s_{j - 1} }}{{q_{j} }},\,\,j > 1. \hfill \\ \end{gathered} \right.$$

Step 4l: The subjective weights of criteria is

18 $$w_{j} = \frac{{s_{j} }}{{\sum\nolimits_{j = 1}^{n} {s_{j} } }},\,\,\forall \,j.$$

Case III: Derive the final weights.

Based on the objective and subjective weights of criteria, an incorporated weighting formula is presented by Eq. (19).

19 $$w_{j} = \gamma w_{j}^{o} + \left( {1 - \gamma } \right)w_{j}^{s} ,\,\,j = 1,2,...,n,$$

wherein $\gamma \in [0, 1]$ is the “*precision objective factor of decision strategy*”.

Step 5: Define “optimal evaluation degree (OED)” of options using Eq. (20).

20 $${\mathbb{R}}_{0} = \left\{ \begin{gathered} \max \,\xi_{ij} ,\,\,\,j \in U_{b} , \hfill \\ \min \,\xi_{ij} ,\,\,\,j\,\, \in U_{c} . \hfill \\ \end{gathered} \right.$$

where $U_{b}$ and $U_{c}$ are the benefit and cost-types of attributes, respectively.

Step 6: Obtain the “weighted NA-IFDM (WNA-IFDM)”.

The WNA-IFDM ${\mathbb{N}}_{w} = \left( {\tilde{\varsigma }_{ij} } \right)_{m\, \times \,n}$ is computed using Eq. (21).

21 $$\tilde{\varsigma }_{ij} = \mathop \oplus \limits_{j = 1}^{n} w_{j} \,\varsigma_{ij} = \left\langle {1 - \prod\limits_{j = 1}^{n} {\left( {1 - b_{ij} } \right)^{{w_{j} }} } ,\prod\limits_{j = 1}^{n} {\left( {n_{ij} } \right)^{{w_{j} }} } \,} \right\rangle ,$$

wherein $\tilde{\varsigma }_{ij} = \left\langle {\tilde{b}_{ij} ,\,\tilde{n}_{ij} } \right\rangle$ is the weighted IFN.

Step 7: Evaluation of IF-Score values.

By using Eq. (4), the score degrees of WNA-IFDM ${\mathbb{N}}_{w} = \left( {\tilde{\varsigma }_{ij} } \right)_{m\, \times \,n}$ are computed as follows:

22 $${\mathbb{S}}^{*} \left( {\tilde{\varsigma }_{ij} } \right) = \left[ {\frac{1}{2}\left( {\left( {\tilde{b}_{ij} - \tilde{n}_{ij} } \right) + 1} \right)} \right].$$

Step 8: Determine the “overall performance rating (OPR)” and “degree of utility (UD)”.

The OPR of each alternative is computed by

23 $${\mathbb{M}}_{i} = \sum\limits_{j = 1}^{n} {{\mathbb{S}}^{*} \left( {\tilde{\varsigma }_{ij} } \right)} ,\,\,\,\forall \,\,i.$$

The highest degree of OPR ${\mathbb{M}}_{i}$ elucidates the more efficient alternative. The preferences of alternatives can be evaluated using Eq. (23). To find the appropriate options, it is not only necessary to obtain the best ranked candidate but also important to assess the relative influence of considered options, in association to the appropriate candidate. The UD of each option is computed by Eq. (24).

24 $${\mathbb{Q}}_{i} = \frac{{{\mathbb{M}}_{i} }}{{{\mathbb{R}}_{0} }};{\mathbb{Q}}_{i} \in \left[ {0,1} \right].$$

Step 9: Estimate the most suitable candidate.

The best alternative can be obtained using

25 $${\mathbb{M}}^{*} = \left\{ {{\mathbb{M}}_{i} |\,\,\mathop {\max }\limits_{i} {\mathbb{Q}}_{i} ;\,\,i = 1\left( 1 \right)m} \right\},$$

wherein ${\mathbb{M}}^{*}$ is most desirable candidate.

## Implementation of proposed method: a case study

In the current portion, the developed methodology is utilized on a case study of “sustainable industrial buildings option (SIBO)” selection in India. In this study, we have taken three real petrochemical projects as alternatives. The considered options are taken to be in diverse settings to assess the impact of location-based indicators (e.g. cultural, migration effects and natural heritage), and these sites are among the large cities of India.

Allocating precise data is a difficult task for DMEs in every MCDM problem^11^. Utilizing IFNs can offer the possibility to tackle ambiguity and uncertainty in DME's decisions. Thus, the DME does not need to allocate specific values to SDIs. Furthermore, the merits of fairly AO are their observation over the preferences and risk attitudes of DMEs. Hence, in this section, a hybrid model is presented that takes the advantages of IFNs and fairly AO to offer a flexible environment for MCDM. As it is obvious the real world is overwhelmed with uncertainty and vague information, in addition the preferences of DMEs during the MCDM process are not just ordinal sorted, but rather influenced by their risk attitudes. Thus, the ideas of incorporation of IFNs to deal with uncertainty and also AOs to consider risk attitudes and preferences were to make the developed framework more compatible with the real world situations.

To assess the recognized SDIs through literature and perspectives of highly experienced and DMEs in differently located yet significant petrochemical plants of the nation, a questionnaire survey was conducted. Questionnaire permits to reach views and attitudes from a certain respondents as a sample with a quantitative assessment^11^. The final determination is to allocate the significance levels to the SDIs to be accordingly usable for the MCDM procedure.

In order to validate the suitability on the SDIs' list and to make sure that the recognized SDIs are practically applicable, a pilot survey was carried out through semi-structured interviews with six experts involved in petrochemical projects in India. Without any guidance, they presented some definite expertise requests for qualifying of DMEs using a panel, which are applied for determination and qualification of following DMEs, in this study: (a) a professor of founding benchmarks and frameworks with 25 years of experience; (b) Environment, Health and Safety (EHS) expert and operations manager with 20 years of experience; (c) administrator of petrochemical projects assessment with 15 years of experience; (d) financial manager with 20 years of experience. Further, the questionnaire is designed to conduct face to face interview with DME panel, as implemented in first round of survey. In this way, DMEs were able to mention their thoughts, accurate possible errors, and check the compatibility of SDIs with the recent concerns. The outcomes of survey certified that the recognized SDIs are to a high degree compatible with the issues, and no conflicting views received from the DME panel, consequently, execution the second round of survey was not required.

The questionnaire was circulated among 25 professionals, comprising researchers, contractors, and consultant firms with minimum 05 years of experience of petrochemical projects in India. The customary approach to define the participants views related to the significance ratings is the 11-point Likert scale. Though, the views of participants can be imprecise and subjective and the similar words can be observed separately and diverse by the participants due to vagueness. Thus, utilizing crisp numbers are not appropriate to explain the LVs and views addressing significance ratings. In order to treat with the vagueness and uncertainty, IFNs are used to express the linguistic significance ratings and given in Tables 2 and 3. Thus, the relative significance of SDIs is examined and defined using the Likert scale.

**Table 2:** Performance rating of DMEs in form of LVs.

LVs	IFNs
Highly considerable (HC)	(0.90, 0.10)
Very considerable (VC)	(0.80, 0.15)
Considerable (C)	(0.70, 0.25)
Average (A)	(0.50, 0.45)
Inconsiderable (I)	(0.40, 0.55)
Very inconsiderable (VI)	(0.20, 0.75)
Extremely inconsiderable (EI)	(0.10, 0.90)

**Table 3:** LVs for performance ranking of options.

LVs	IFNs
Completely satisfactory (CS)	(0.95, 0.05)
Highly satisfactory (HS)	(0.85, 0.10)
Very satisfactory (VS)	(0.80, 0.15)
Satisfactory (S)	(0.70, 0.20)
Slight satisfactory (SS)	(0.60, 0.30)
Fair (F)	(0.50, 0.40)
Slight unsatisfactory (SU)	(0.40, 0.50)
Unsatisfactory (U)	(0.30,0.60)
Very unsatisfactory (VU)	(0.20, 0.70)
Highly unsatisfactory (HU)	(0.10, 0.80)
Entirely unsatisfactory (EU)	(0.05, 0.95)

For this evaluation process, a set of four DMEs, who are all very skilled in the selected region. Suppose that it is not promising to assess and associate all the projects exactly using the whole recognized SDIs (Table 1). Consequently, the assessment of options were done using a limited number of recognized SDIs, though, the SDIs were the similar for considered options. Here, we choose 27 SDIs (Table 1) based on operation stage of project life cycle. The DME's opinions and the accessible information on SDIs are the key parameters in this evaluation process. Here, we implement the proposed method on SIBOs assessment problem.

For this case study, Table 2 presents the “linguistic values (LVs)” and the corresponding IFNs for the SDIs and the IBs evaluation. Table 3 illustrates the LVs by DMEs for the criteria of considered evaluation process. Using Eq. (8) and Table 2, the weight of DMEs are obtained and presented in Table 4. For SIBO assessment, we obtain the LDM $Z^{\left( k \right)} = \left( {\xi_{ij}^{\left( k \right)} } \right)_{m\, \times \,n}$ for each DME and are shown in Table 5. Using Eq. (6), Eq. (9) and Table 5, the A-IFDM for SIBOs assessment is computed according to the DMEs’ opinions given in Table 6.

**Table 4:** DMEs’ weights for SIBO selection.

DMEs	$t_{1}$	$t_{2}$	$t_{3}$	$t_{4}$
LVs	VC(0.80, 0.15)	C(0.70, 0.25)	A(0.50, 0.45)	I(0.40, 0.55)
DMEs Weight	0.333	0.2917	0.2083	0.1667

**Table 5:** LDM for SIBO selection.

	$M_{1}$	$M_{2}$	$M_{3}$
*U*_1_	(SU,VU,VU,F)	(F,SU,VU,U)	(S,SS,F,F)
*U*_2_	(U,U,F,SS)	(U,VU,VU,SU)	(S,F,SS,F)
*U*_3_	(S,VS,S,S)	(S,VS,VS,S)	(SU,F,S,F)
*U*_4_	(SU,F,S,SS)	(VS,F,S,S)	(SU,SU,SS,F)
*U*_5_	(SU,SS,S,F)	(SU,S,S,VS)	(SS,SU,F,F)
*U*_6_	(VU,SU,U,VU)	(F,VU,U,VU)	(VS,S,F,SU)
*U*_7_	(SU,SS,U,F)	(U,VU,U,VU)	(F,SS,F,VS)
*U*_8_	(S,VS,VS,SS)	(F,S,VS,VS)	(SU,F,SS,VU)
*U*_9_	(SU,U,U,F)	(F,VS,S,S)	(F,SU,S,F)
*U*_10_	(U,VS,S,F)	(SU,SS,S,VS)	(F,SS,SU,F)
*U*_11_	(SU,U,U,VU)	(SS,U,U,VU)	(SS,S,F,U)
*U*_12_	(S,F,S,SS)	(SU,F,SU,VU)	(S,VS,F,SU)
*U*_13_	(SS,F,S,VS)	(F,SS,S,VS)	(SU,SS,SU,F)
*U*_14_	(SU,F,S,SS)	(SU,SS,S,VS)	(SU,VS,SS,F)
*U*_15_	(VS,SS,F,F)	(SU,VS,S,SS)	(F,SU,VS,F)
*U*_16_	(SU,U,VU,U)	(F,U,VU,VU)	(SS,F,VS,F)
*U*_17_	(SU,SU,F,F)	(U,VU,SU,VU)	(SS,SU,F,SS)
*U*_18_	(SS,VS,S,VS)	(F,VS,S,VS)	(F,VU,U,F)
*U*_19_	(SU,F,S,SU)	(SS,VS,S,SU)	(SU,SS,F,SU)
*U*_20_	(SS,F,S,F)	(F,SS,F,VS)	(SU,VU,F,VU)
*U*_21_	(U,F,VU,SU)	(VU,U,SU,SU)	(SS,S,S,U)
*U*_22_	(SU,U,VU,F)	(U,VU,VU,F)	(F,SS,VU,S)
*U*_23_	(SS,VS,F,S)	(S,VS,SS,S)	(SS,F,SU,U)
*U*_24_	(SU,F,S,SS)	(F,S,F,S)	(S,F,SU,U)
*U*_25_	(SU,U,VU,VU)	(F,SU,S,VU)	(SU,SS,U,F)
*U*_26_	(U,VU,F,VU)	(F,SS,SU,U)	(F,S,SS,S)
*U*_27_	(F,VS,S,F)	(F,SU,F,F)	(U,VU,SU,VU)

**Table 6:** The A-IFDM for SIBOs evaluation.

	$M_{1}$	$M_{2}$	$M_{3}$
*U*_1_	(0.306, 0.594)	(0.368, 0.532)	(0.602, 0.298)
*U*_2_	(0.389, 0.511)	(0.261, 0.639)	(0.594, 0.306)
*U*_3_	(0.733, 0.185)	(0.755, 0.175)	(0.515, 0.385)
*U*_4_	(0.532, 0.368)	(0.689, 0.231)	(0.459, 0.441)
*U*_5_	(0.544, 0.356)	(0.635, 0.276)	(0.506, 0.394)
*U*_6_	(0.272, 0.628)	(0.310, 0.590)	(0.661, 0.260)
*U*_7_	(0.455, 0.445)	(0.251, 0.649)	(0.589, 0.322)
*U*_8_	(0.741, 0.188)	(0.687, 0.236)	(0.433, 0.467)
*U*_9_	(0.365, 0.535)	(0.677, 0.241)	(0.519, 0.381)
*U*_10_	(0.583, 0.335)	(0.602, 0.309)	(0.510, 0.390)
*U*_11_	(0.313, 0.587)	(0.378, 0.522)	(0.566, 0.334)
*U*_12_	(0.719, 0.199)	(0.391, 0.514)	(0.656, 0.259)
*U*_13_	(0.635, 0.275)	(0.632, 0.279)	(0.477, 0.423)
*U*_14_	(0.532, 0.368)	(0.602, 0.309)	(0.592, 0.326)
*U*_15_	(0.644, 0.277)	(0.632, 0.287)	(0.546, 0.368)
*U*_16_	(0.308, 0.592)	(0.319, 0.581)	(0.607, 0.307)
*U*_17_	(0.372, 0.528)	(0.269, 0.631)	(0.523, 0.377)
*U*_18_	(0.722, 0.205)	(0.696, 0.231)	(0.362, 0.538)
*U*_19_	(0.498, 0.402)	(0.661, 0.257)	(0.481, 0.419)
*U*_20_	(0.580, 0.320)	(0.589, 0.322)	(0.318, 0.582)
*U*_21_	(0.347, 0.553)	(0.298, 0.602)	(0.610, 0.290)
*U*_22_	(0.340, 0.560)	(0.275, 0.625)	(0.500, 0.400)
*U*_23_	(0.667, 0.252)	(0.715, 0.203)	(0.480, 0.420)
*U*_24_	(0.532, 0.368)	(0.509, 0.391)	(0.522, 0.378)
*U*_25_	(0.289, 0.611)	(0.464, 0.436)	(0.455, 0.445)
*U*_26_	(0.287, 0.613)	(0.476, 0.424)	(0.620, 0.280)
*U*_27_	(0.645, 0.273)	(0.471, 0.429)	(0.269, 0.631)

Since the SDIs *U*_1_, *U*_2_, *U*_3_, *U*_9_, *U*_11_, *U*_22_, *U*_25_ and *U*_26_ are cost-type and the rest of all are benefit-type. Hence, using Eq. (10), the NA-IFDM is obtained in Table 7.

**Table 7:** The NA-IFDM for SIBOs assessment.

Indicators	$M_{1}$	$M_{2}$	$M_{3}$
*U*_1_	(0.594, 0.306)	(0.532, 0.368)	(0.298, 0.602)
*U*_2_	(0.511, 0.389)	(0.639, 0.261)	(0.306, 0.594)
*U*_3_	(0.185, 0.733)	(0.175, 0.755)	(0.385, 0.515)
*U*_4_	(0.532, 0.368)	(0.689, 0.231)	(0.459, 0.441)
*U*_5_	(0.544, 0.356)	(0.635, 0.276)	(0.506, 0.394)
*U*_6_	(0.272, 0.628)	(0.310, 0.590)	(0.661, 0.260)
*U*_7_	(0.455, 0.445)	(0.251, 0.649)	(0.589, 0.322)
*U*_8_	(0.741, 0.188)	(0.687, 0.236)	(0.433, 0.467)
*U*_9_	(0.535, 0.365)	(0.241, 0.677)	(0.381, 0.519)
*U*_10_	(0.583, 0.335)	(0.602, 0.309)	(0.510, 0.390)
*U*_11_	(0.587, 0.313)	(0.522, 0.378)	(0.334, 0.566)
*U*_12_	(0.719, 0.199)	(0.391, 0.514)	(0.656, 0.259)
*U*_13_	(0.635, 0.275)	(0.632, 0.279)	(0.477, 0.423)
*U*_14_	(0.532, 0.368)	(0.602, 0.309)	(0.592, 0.326)
*U*_15_	(0.644, 0.277)	(0.632, 0.287)	(0.546, 0.368)
*U*_16_	(0.308, 0.592)	(0.319, 0.581)	(0.607, 0.307)
*U*_17_	(0.372, 0.528)	(0.269, 0.631)	(0.523, 0.377)
*U*_18_	(0.722, 0.205)	(0.696, 0.231)	(0.362, 0.538)
*U*_19_	(0.498, 0.402)	(0.661, 0.257)	(0.481, 0.419)
*U*_20_	(0.580, 0.320)	(0.589, 0.322)	(0.318, 0.582)
*U*_21_	(0.347, 0.553)	(0.298, 0.602)	(0.610, 0.290)
*U*_22_	(0.560, 0.340)	(0.625, 0.275)	(0.400, 0.500)
*U*_23_	(0.667, 0.252)	(0.715, 0.203)	(0.480, 0.420)
*U*_24_	(0.532, 0.368)	(0.509, 0.391)	(0.522, 0.378)
*U*_25_	(0.611, 0.289)	(0.436, 0.464)	(0.445, 0.455)
*U*_26_	(0.613, 0.287)	(0.424, 0.476)	(0.280, 0.620)
*U*_27_	(0.645, 0.273)	(0.471, 0.429)	(0.269, 0.631)

In order to derive the objective weights of SDIs, the A-IFDM is normalized by using Eq. (10). Then, the overall performances of the SIBOs are computed by Eq. (11), therefore, we have *S*_1_ = 0.450, *S*_2_ = 0.504, and *S*_3_ = 0.534. With the use of Eqs. (13)–(15), the remaining computational steps of MEREC are determined and given in Table 8.

**Table 8:** Objective weights for SDIs using IF-MEREC.

Indicators	$\left( {S_{ij}^{^{\prime}} } \right)$ values			*V*_*j*_	$w_{j}^{o}$
	*M*_1_	*M*_2_	*M*_3_		
*U*_1_	0.439	0.492	0.511	0.046	0.0388
*U*_2_	0.436	0.496	0.511	0.045	0.0379
*U*_3_	0.414	0.469	0.516	0.090	0.0757
*U*_4_	0.437	0.497	0.519	0.035	0.0294
*U*_5_	0.437	0.495	0.521	0.034	0.0287
*U*_6_	0.422	0.481	0.526	0.058	0.0491
*U*_7_	0.433	0.477	0.524	0.053	0.0452
*U*_8_	0.444	0.497	0.518	0.029	0.0246
*U*_9_	0.437	0.475	0.515	0.060	0.0507
*U*_10_	0.438	0.494	0.521	0.034	0.0285
*U*_11_	0.439	0.492	0.513	0.044	0.0374
*U*_12_	0.443	0.485	0.526	0.033	0.0279
*U*_13_	0.440	0.495	0.520	0.032	0.0270
*U*_14_	0.439	0.495	0.522	0.032	0.0272
*U*_15_	0.441	0.495	0.522	0.030	0.0250
*U*_16_	0.425	0.482	0.525	0.056	0.0478
*U*_17_	0.429	0.478	0.522	0.059	0.0496
*U*_18_	0.443	0.497	0.514	0.033	0.0279
*U*_19_	0.435	0.496	0.520	0.036	0.0305
*U*_20_	0.439	0.494	0.512	0.043	0.0365
*U*_21_	0.428	0.480	0.525	0.055	0.0465
*U*_22_	0.438	0.495	0.516	0.038	0.0322
*U*_23_	0.441	0.498	0.520	0.028	0.0240
*U*_24_	0.437	0.491	0.522	0.038	0.0323
*U*_25_	0.440	0.488	0.519	0.041	0.0351
*U*_26_	0.440	0.487	0.510	0.051	0.0431
*U*_27_	0.441	0.489	0.509	0.049	0.0412

Table 9 illustrates the LVs by DMEs for the criteria significances. Based on Definition 3.2, the score values of corresponding aggregated IFNs are computed in Table 9.

**Table 9:** Score values of SDIs.

Indicators	$t_{1}$	$t_{2}$	$t_{3}$	$t_{4}$	Aggregated IFNs	Score values
*U*_1_	F	SU	SS	SU	(0.481, 0.417, 0.102)	0.532
*U*_2_	SS	F	F	SS	(0.553, 0.346, 0.101)	0.603
*U*_3_	VU	U	SU	U	(0.291, 0.608, 0.101)	0.342
*U*_4_	F	F	F	U	(0.471, 0.428, 0.101)	0.522
*U*_5_	VU	VU	VU	SU	(0.237, 0.662, 0.101)	0.288
*U*_6_	VS	SS	S	S	(0.715, 0.205, 0.080)	0.755
*U*_7_	S	F	F	S	(0.613, 0.283, 0.104)	0.665
*U*_8_	SS	VS	VU	U	(0.586, 0.328, 0.086)	0.629
*U*_9_	S	SU	SU	U	(0.472, 0.416, 0.112)	0.528
*U*_10_	VS	S	F	F	(0.683, 0.236, 0.082)	0.723
*U*_11_	F	U	VU	U	(0.357, 0.541, 0.102)	0.408
*U*_12_	VS	SU	F	SS	(0.626, 0.293, 0.081)	0.666
*U*_13_	S	SS	SU	U	(0.566, 0.327, 0.107)	0.619
*U*_14_	VS	S	U	SU	(0.649, 0.266, 0.085)	0.691
*U*_15_	SS	SS	SU	U	(0.522, 0.375, 0.103)	0.574
*U*_16_	SS	SU	S	U	(0.524, 0.369, 0.107)	0.578
*U*_17_	VU	U	U	SU	(0.287, 0.613, 0.101)	0.337
*U*_18_	VS	SU	SU	U	(0.573, 0.345, 0.082)	0.614
*U*_19_	S	F	U	VU	(0.511, 0.379, 0.110)	0.566
*U*_20_	VS	SU	F	S	(0.643, 0.274, 0.083)	0.684
*U*_21_	SS	F	U	VU	(0.462, 0.434, 0.104)	0.514
*U*_22_	F	SS	SU	U	(0.485, 0.412, 0.102)	0.537
*U*_23_	SU	F	VU	U	(0.380, 0.518, 0.102)	0.431
*U*_24_	SS	F	F	U	(0.509, 0.389, 0.102)	0.560
*U*_25_	SU	SU	F	VU	(0.394, 0.505, 0.101)	0.445
*U*_26_	VU	F	SU	SU	(0.631, 0.288, 0.081)	0.672
*U*_27_	SS	SU	U	S	(0.504, 0.388, 0.108)	0.558

Using Eqs. (16)–(18) and Table 9, the subjective weight of SDIs is presented in Table 10 and derived as

$$w_{j}^{s} \, = \,\left( {0.0{359}, \, 0.0{385}, \, 0.0{298}, \, 0.0{355}, \, 0.0{283}, \, 0.0{447}, \, 0.0{4}0{9}, \, 0.0{395}, \, 0.0{357}, \, 0.0{433}, \, 0.0{318}, \, 0.0{41}0, \, 0.0{391}, \, 0.0{42}0, \, 0.0{374}, \, 0.0{376}, \, 0.0{297}, \, 0.0{389}, \, 0.0{371}, \, 0.0{417}, \, 0.0{352}, \, 0.0{36}0, \, 0.0{325}, \, 0.0{369}, \, 0.0{33}0, \, 0.0{412}, \, 0.0{368}} \right).$$

**Table 10:** Subjective weights for SDIs using the SWARA method.

Indicators	Score values	Relative significance of SDI values	Relative degree	Recalculated weight	Final weight
*U*_6_	0.755	–	1.000	1.000	0.0447
*U*_10_	0.723	0.032	1.032	0.969	0.0433
*U*_14_	0.691	0.032	1.032	0.939	0.0420
*U*_20_	0.684	0.007	1.007	0.932	0.0417
*U*_26_	0.672	0.012	1.012	0.921	0.0412
*U*_12_	0.666	0.006	1.006	0.916	0.0410
*U*_7_	0.665	0.001	1.001	0.915	0.0409
*U*_8_	0.629	0.036	1.036	0.883	0.0395
*U*_13_	0.619	0.010	1.010	0.874	0.0391
*U*_18_	0.614	0.005	1.005	0.870	0.0389
*U*_2_	0.603	0.011	1.011	0.861	0.0385
*U*_16_	0.578	0.025	1.025	0.840	0.0376
*U*_15_	0.574	0.004	1.004	0.837	0.0374
*U*_19_	0.566	0.008	1.008	0.830	0.0371
*U*_24_	0.560	0.006	1.006	0.825	0.0369
*U*_27_	0.558	0.002	1.002	0.823	0.0368
*U*_22_	0.537	0.021	1.021	0.806	0.0360
*U*_1_	0.532	0.005	1.005	0.802	0.0359
*U*_9_	0.528	0.004	1.004	0.799	0.0357
*U*_4_	0.522	0.006	1.006	0.794	0.0355
*U*_21_	0.514	0.008	1.008	0.788	0.0352
*U*_25_	0.445	0.069	1.069	0.737	0.0330
*U*_23_	0.431	0.014	1.014	0.727	0.0325
*U*_11_	0.408	0.023	1.023	0.711	0.0318
*U*_3_	0.342	0.066	1.066	0.667	0.0298
*U*_17_	0.337	0.005	1.005	0.664	0.0297
*U*_5_	0.288	0.049	1.049	0.633	0.0283

By combining the objective and subjective weights of SDIs, an integrated weight of SDIs ($\tau =0.5$) are shown as below:

$$w_{j} = \,\left( {0.0{373}, \, 0.0{382}, \, 0.0{528}, \, 0.0{324}, \, 0.0{285}, \, 0.0{469}, \, 0.0{431}, \, 0.0{321}, \, 0.0{432}, \, 0.0{359}, \, 0.0{346}, \, 0.0{344}, \, 0.0{331}, \, 0.0{346}, \, 0.0{312}, \, 0.0{427}, \, 0.0{396}, \, 0.0{334}, \, 0.0{338}, \, 0.0{391}, \, 0.0{4}0{9}, \, 0.0{341}, \, 0.0{282}, \, 0.0{346}, \, 0.0{34}0, \, 0.0{421}, \, 0.0{39}0} \right).$$

The optimal performance rating $\left( {{\mathbb{R}}_{0} } \right)$ of each SIBO is computed using Eq. (20). The obtained OPRs of SIBOs are represented in Table 11.

**Table 11:** The OPR of sustainable industrial building options.

Criteria	*U*_1_	*U*_2_	*U*_3_	*U*_4_	*U*_5_
${\mathbb{R}}_{0}$	(0.306, 0.594)	(0.261, 0.639)	(0.515, 0.385)	(0.689, 0.231)	(0.635, 0.276)
*U*_6_	*U*_7_	*U*_8_	*U*_9_	*U*_10_	*U*_11_
(0.661, 0.260)	(0.589, 0.322)	(0.741, 0.188)	(0.365, 0.535)	(0.602, 0.309)	(0.313, 0.587)
*U*_12_	*U*_13_	*U*_14_	*U*_15_	*U*_16_	*U*_17_
(0.719, 0.199)	(0.635, 0.275)	(0.602, 0.309)	(0.644, 0.277)	(0.607, 0.307)	(0.523, 0.377)
*U*_18_	*U*_19_	*U*_20_	*U*_21_	*U*_22_	*U*_23_
(0.722, 0.205)	(0.661, 0.257)	(0.580, 0.320)	(0.610, 0.290)	(0.275, 0.625)	(0.715, 0.203)
*U*_24_	*U*_25_	*U*_26_	*U*_27_		
(0.532, 0.368)	(0.289, 0.611)	(0.287, 0.613)	(0.645, 0.273)		

Based on Eq. (21) and using Table 7, the WNA-IFDM for SIBOs selection is formed and given in Table 12.

**Table 12:** The WNA-IFDM for SIBOs assessment.

Indicators	${\mathbb{R}}_{0}$	$M_{1}$	$M_{2}$	$M_{3}$
*U*_1_	(0.033, 0.957)	(0.033, 0.957)	(0.028, 0.963)	(0.013, 0.981)
*U*_2_	(0.038, 0.950)	(0.027, 0.965)	(0.038, 0.950)	(0.014, 0.980)
*U*_3_	(0.025, 0.966)	(0.011, 0.984)	(0.010, 0.985)	(0.025, 0.966)
*U*_4_	(0.037, 0.954)	(0.024, 0.968)	(0.037, 0.954)	(0.020, 0.974)
*U*_5_	(0.028, 0.964)	(0.022, 0.971)	(0.028, 0.964)	(0.020, 0.974)
*U*_6_	(0.049, 0.939)	(0.015, 0.978)	(0.017, 0.976)	(0.049, 0.939)
*U*_7_	(0.038, 0.952)	(0.026, 0.966)	(0.012, 0.982)	(0.038, 0.952)
*U*_8_	(0.042, 0.948)	(0.042, 0.948)	(0.037, 0.955)	(0.018, 0.976)
*U*_9_	(0.033, 0.957)	(0.033, 0.957)	(0.012, 0.983)	(0.021, 0.972)
*U*_10_	(0.033, 0.959)	(0.031, 0.961)	(0.033, 0.959)	(0.025, 0.967)
*U*_11_	(0.030, 0.961)	(0.030, 0.961)	(0.025, 0.967)	(0.014, 0.980)
*U*_12_	(0.043, 0.946)	(0.043, 0.946)	(0.017, 0.977)	(0.036, 0.955)
*U*_13_	(0.033, 0.958)	(0.033, 0.958)	(0.032, 0.959)	(0.021, 0.972)
*U*_14_	(0.031, 0.960)	(0.026, 0.966)	(0.031, 0.960)	(0.031, 0.962)
*U*_15_	(0.032, 0.961)	(0.032, 0.961)	(0.031, 0.962)	(0.024, 0.969)
*U*_16_	(0.039, 0.951)	(0.016, 0.978)	(0.016, 0.977)	(0.039, 0.951)
*U*_17_	(0.029, 0.962)	(0.018, 0.975)	(0.012, 0.982)	(0.029, 0.962)
*U*_18_	(0.042, 0.948)	(0.042, 0.948)	(0.039, 0.952)	(0.015, 0.980)
*U*_19_	(0.036, 0.955)	(0.023, 0.970)	(0.036, 0.955)	(0.022, 0.971)
*U*_20_	(0.033, 0.956)	(0.033, 0.956)	(0.034, 0.957)	(0.015, 0.979)
*U*_21_	(0.038, 0.951)	(0.017, 0.976)	(0.014, 0.979)	(0.038, 0.951)
*U*_22_	(0.033, 0.957)	(0.028, 0.964)	(0.033, 0.957)	(0.017, 0.977)
*U*_23_	(0.035, 0.956)	(0.031, 0.962)	(0.035, 0.956)	(0.018, 0.976)
*U*_24_	(0.026, 0.966)	(0.026, 0.966)	(0.024, 0.968)	(0.025, 0.967)
*U*_25_	(0.032, 0.959)	(0.032, 0.959)	(0.019, 0.974)	(0.020, 0.974)
*U*_26_	(0.039, 0.949)	(0.039, 0.949)	(0.023, 0.969)	(0.014, 0.980)
*U*_27_	(0.040, 0.951)	(0.040, 0.951)	(0.025, 0.968)	(0.012, 0.982)

From Table 12 and Eq. (22), the score degrees ${\mathbb{S}}^{*} \left( {\tilde{\varsigma }_{ij} } \right)$ of SIBOs are presented in Table 13. Corresponding to Eq. (23)-Eq. (24), the OPR $\left( {{\mathbb{M}}_{i} } \right)$ and UD $\left( {{\mathbb{Q}}_{i} } \right)$ of each SIBO is calculated and specified in Table 13. Then, from Eq. (25), the prioritization of the SIBOs is determined as $M_{1} \succ M_{2} \succ M_{3} .$ Hence, the desirable alternative building is $M_{1} .$

**Table 13:** The Score value, OPR and UD for SIBOs assessment.

Indicators	${\mathbb{R}}_{0}$	$M_{1}$	$M_{2}$	$M_{3}$
*U*_1_	0.038	0.038	0.032	0.016
*U*_2_	0.044	0.031	0.044	0.017
*U*_3_	0.030	0.013	0.012	0.030
*U*_4_	0.042	0.028	0.042	0.023
*U*_5_	0.032	0.026	0.032	0.023
*U*_6_	0.055	0.018	0.021	0.055
*U*_7_	0.043	0.030	0.015	0.043
*U*_8_	0.047	0.047	0.041	0.021
*U*_9_	0.038	0.038	0.014	0.024
*U*_10_	0.037	0.035	0.037	0.029
*U*_11_	0.035	0.035	0.029	0.017
*U*_12_	0.048	0.048	0.020	0.041
*U*_13_	0.037	0.037	0.037	0.025
*U*_14_	0.036	0.030	0.036	0.034
*U*_15_	0.035	0.035	0.034	0.028
*U*_16_	0.044	0.019	0.020	0.044
*U*_17_	0.033	0.022	0.015	0.033
*U*_18_	0.047	0.047	0.043	0.018
*U*_19_	0.040	0.027	0.040	0.025
*U*_20_	0.038	0.038	0.039	0.018
*U*_21_	0.044	0.021	0.017	0.044
*U*_22_	0.038	0.032	0.038	0.020
*U*_23_	0.039	0.034	0.039	0.021
*U*_24_	0.030	0.030	0.028	0.029
*U*_25_	0.037	0.036	0.023	0.023
*U*_26_	0.045	0.045	0.027	0.017
*U*_27_	0.044	0.044	0.029	0.015
OMR	1.078	0.886	0.805	0.733
Degree of utility	1.000	0.8218	0.7472	0.6800

### Comparative study

Comparison with existing studies is presented to certify the outcomes of introduced ARAS approach. In this respect, we have chosen the previously developed methods which are IF-COPRAS^81^ and IF-WASPAS^36^.

#### IF-COPRAS model

Steps 1–4: Same as previous method.

Step 5: Sum of the degrees of criteria for benefit and cost-type.

In this step, each option is signified with its addition of maximizing criterion $\alpha_{i} ,$ which is assigned to benefit-type, and minimizing criterion $\beta_{i} ,$ which is assigned to cost-type using

26 $$\alpha_{i} = \mathop \oplus \limits_{j = 1}^{l} \,w_{j} \,\overline{\xi }_{ij} ,\,\,\,\,i = 1\left( 1 \right)m.$$

27 $$\beta_{i} = \mathop \oplus \limits_{j = l + 1}^{n} \,w_{j} \,\overline{\xi }_{ij} ,\,\,\,i = 1\left( 1 \right)m.$$

Here, $l$ is the number of benefit-type and *n* is the total number of criteria.

Step 6: Calculate the “relative degree (RD)” of each option.

The RD $\left( {\gamma_{i} } \right)$ of *i*th option is obtained using

28 $$\gamma_{i} = {\mathbb{S}}^{*} \left( {\alpha_{i} } \right) + \frac{{\mathop {\min }\limits_{i} {\mathbb{S}}^{*} \left( {\beta_{i} } \right)\sum\limits_{i = 1}^{m} {{\mathbb{S}}^{*} \left( {\beta_{i} } \right)} }}{{{\mathbb{S}}^{*} \left( {\beta_{i} } \right)\sum\limits_{i = 1}^{m} {\frac{{\mathop {\min }\limits_{i} {\mathbb{S}}^{*} \left( {\beta_{i} } \right)}}{{{\mathbb{S}}^{*} \left( {\beta_{i} } \right)}}} }}.$$

Here, ${\mathbb{S}}^{*} \left( {\alpha_{i} } \right)$ and ${\mathbb{S}}^{*} \left( {\beta_{i} } \right)$ denote the score values of $\alpha_{i}$ and $\beta_{i} ,$ respectively.

Step 7: Define the “priority order (PO)” of the options.

Corresponding to the RD, the PO of the options is obtained. The maximum RD of option has been ranked as superior significance, and thus, it is the most appropriate option.

29 $$E^{*} = \,\,\mathop {\max }\limits_{i} \,\gamma_{i} ,\,\,\,\forall \,\,i.$$

Step 8: Compute the “utility degree (UD)” of each option.

By evaluating the examined options with the optimal one, the UD of each option is calculated based on Eq. (30).

30 $$\delta_{i} = \frac{{\gamma_{i} }}{{\,\,\gamma_{\max } }} \times 100\,\% ,\,\,i = 1\left( 1 \right)m.$$

Now, the results IF-COPRAS^81^ are described in Table 14. From Table 6 and Eqs. (26)–(30), $\alpha_{i} ,$ $\beta_{i} ,$ RD and UD of each SIBO are evaluated. According to UD, $M_{1}$ has been found to be the best SIBO since it has the maximum RD (0.546).

**Table 14:** The results of IF-COPRAS for SIBO assessment.

SIBO	$\alpha_{i}$	${\mathbb{S}}^{*} \left( {\alpha_{i} } \right)$	$\beta_{i}$	${\mathbb{S}}^{*} \left( {\beta_{i} } \right)$	$\gamma_{i}$	$\delta_{i}$
$M_{1}$	(0.446, 0.492)	0.477	(0.160, 0.789)	0.186	0.546	100.00%
$M_{2}$	(0.415, 0.510)	0.452	(0.203, 0.743)	0.230	0.544	99.75%
$M_{3}$	(0.417, 0.519)	0.449	(0.223, 0.717)	0.253	0.522	95.73%

#### IF-WASPAS model

Steps 1–4: Follow the developed model.

Step 5: Estimate the “weighted sum measure (WSM)” and the “weighted product measure (WPM)” using the following expressions:

31 $$\Im_{i}^{(1)} \, = \,\mathop \oplus \limits_{j = 1}^{n} w_{j} \,\varsigma_{ij} .$$

32 $$\Im_{i}^{(2)} \, = \,\mathop \otimes \limits_{j = 1}^{n} w_{j} \,\varsigma_{ij} ,\,\,i = 1,2,...,m.$$

Step 6: Find the measure of “weighted aggregated sum product assessment (WASPAS)” using Eq. (33).

33 $$Q_{i} \, = \,\varepsilon \,\Im_{i}^{(1)} \, + \,\left( {1 - \,\varepsilon } \right)\,\Im_{i}^{(2)} ,\,\,i = 1,2,...,m,$$

where $\hbar \in [0,1][0, 1]$ means strategic coefficient.

Step 7: Prioritize the options with the score value of $Q_{i} .$

Using Eqs. (31)–(33), the whole procedures of IF-WASPAS are computed and demonstrated in Table 15.

**Table 15:** The IF-WASPAS method for prioritizing SIBOs.

Options	$\wp_{i}^{(1)} \,$	$\wp_{i}^{(2)} \,$	${\mathbb{S}}\left( {\wp_{i}^{(1)} \,} \right)$	${\mathbb{S}}\left( {\wp_{i}^{(2)} } \right)\,$	$Q_{i} \left( \hbar \right)$	_Ranks_
*M*_1_	(0.540, 0.360, 0.099)	(0.497, 0.404, 0.099)	0.590	0.547	0.5683	1
*M*_2_	(0.505, 0.395, 0.099)	(0.447, 0.455, 0.098)	0.555	0.496	0.5255	2
*M*_3_	(0.463, 0.428, 0.109)	(0.444, 0.456, 0.100)	0.517	0.494	0.5057	3

Therefore, the ranking of SIBOs is $M_{1} \succ M_{2} \succ M_{3}$ and according to UD, $M_{1}$ has been found to be the best SIBO among set of given three alternatives.

**Figure 3:**
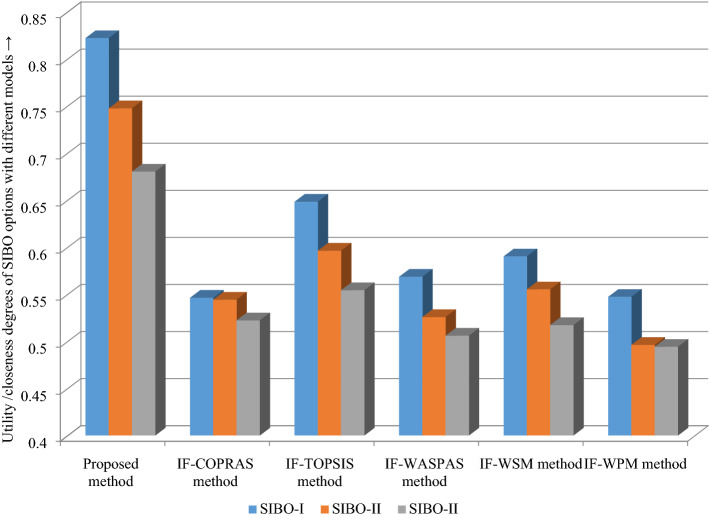
Assessment degree of options for prioritizing SIBO with different methods.

To validate the effectiveness of the developed methodology, we also compare it with the IF-TOPSIS^82^ in Table 16. The outcomes of the developed IF-MEREC-SWARA-ARAS framework with extant tools are given in Table 16 and Fig. 3. It can be observed from different parameter viewpoints that the proposed methodology is certainly a novel contribution as it combines all the key aspects of the MCDM procedure for treating the problems within uncertain settings.

**Table 16:** Parameters to compare the diverse approaches.

Aspects	Boran et al.^82^ method	Gitinavard and Shirazi^81^ method	Mishra et al.^36^ method	Introduced approach
Standards	IF-TOPSIS method	IF-COPRAS method	IF-WASPASmethod based on similarity measures	IF-ARAS method based on IF-MEREC-SWARA method
Aggregation process	Arithmetic, geometric	Arithmetic, geometric	Arithmetic, geometric	Fairly aggregation operators
Criteria weights	Computed (IFWAO)	Computed (IFWAO)	Computed (proposed similarity measures)	Computed (IF-MEREC-SWARA method)
MCDM procedure	Group	Group	Group	Group
HD in evaluations	Included	Excluded	Excluded	Included
DME weights	Evaluated (Score function based method)	Evaluated (IFWGO)	Not Applicable	Evaluated (Score value-based procedure)
Normalization type	Linear	Not Applicable	Linear	Linear
Ranking order	$M_{1} \succ M_{2} \succ M_{3}$	$M_{1} \succ M_{2} \succ M_{3}$	$M_{1} \succ M_{2} \succ M_{3}$	$M_{1} \succ M_{2} \succ M_{3}$
Optimal SIBOs option	$M_{1}$	$M_{1}$	$M_{1}$	$M_{1}$

The key benefits of the developed IF-MEREC-SWARA-ARAS methodology are discussed as follows:

- The developed IF-MEREC-SWARA-ARAS technique evades the defuzzification and employs the core operations of IFNs through the evaluation and ranking process.
- In IF-TOPSIS^82^, it is necessary to calculate the distances between each assessment of options by means of considered criteria and that of the ideal solution, which is time-consuming and decreases the accuracy of the results. While, the calculation process of the IF-MEREC-SWARA-ARAS method is simpler, and thus the accuracy and reliability of the results are higher.
- The proposed method utilizes the fairly aggregation operators for aggregating the individual decision information, which avoids the drawbacks of existing operators used by Boran et al.^82^, Gitinavard and Shirazi^81^, and Mishra et al.^36^.
- In Boran et al.^82^ and proposed study, the weights of DMEs are obtained using score function based method ensuing in more accurate individual measure for determining the DMEs’ weights unlike randomly preferred DMEs’ weights in Mishra et al.^36^.
- To handle the vagueness that appears in MCDM problems, all input variables, i.e., the predictions of options on criteria by several DMEs, DMEs’ weights by the experts, and weights of the criteria by DMEs, are considered as uncertain concerns and expressed in the form of IFNs. The HD is measured as significant way in the entire procedure and the desirable option is determined by means of evaluation values of all three inputs parameters.

To sum up, Table 17 suitably signifies the benefits of the developed model in comparison with Boran et al.^82^, Gitinavard and Shirazi^81^ and Mishra et al.^36^ methods. Therefore, Table 17 shows that the developed model, in comparison with Boran et al.^82^, Gitinavard and Shirazi^81^ and Mishra et al.^36^ tools, has the advantages comprising modeling of uncertainty, the weights of criteria, aggregation operators and DMEs’ weights. In this way, the time complexity of the Gitinavard and Shirazi^81^ and Mishra et al.^36^ approaches is lower than the proposed method and Boran et al.^82^ model. Whereas Boran et al.^82^ model has higher time complexity than the developed model and the four methods are suitable with the comparison parameter of support to MCDM. Accordingly, the proposed IF-MEREC-SWARA-ARAS method could be suitable based on its unique features.

**Table 17:** Summarized comparative assessment of the developed model with extant tools.

Comparative parameters	The comparison outcomes
Modeling uncertainty	Due to the consideration of intuitionistic fuzzy information, the four models are suitable to tackle with uncertain settings in SIBO selection problems. Though, the developed model is taken IFSs which could suitably intricate the imprecision and subjectivity in MCDM problems describing membership degree, non-membership degree and indeterminacy degree of an element under a set to lessening errors
Weights of SDIs/criteria	The developed model is computed the integrated weights of SDIs combining the objective and subjective weights based on IF-MEREC and IF-SWARA tools. Thus, the developed model could lead to a precise solution. In contrast, in IF-WASPAS, the criteria weight is obtained with similarity measure-based tool, in IF-TOPSIS, the criteria weight is obtained using IFWA operators and in IF-COPRAS, the criteria weight is chosen randomly, which did not consider a procedure for finding the weights of criteria
Aggregation operator	The developed model proposes fairly AOs to combine to avoid data loss. In some cases, when there are many DMEs employed to judge the candidates, first aggregation could lead to data loss. The Boran et al.^82^, Gitinavard and Shirazi^81^ and Mishra et al.^36^ methods do not consider this concept,therefore, the obtained results from the proposed approach of this study are more reliable
Experts’ weights	The developed model and Boran et al.^82^ model compute the DMEs’ weights based on the IF-score value-based tool to decrease errors. Therefore, the developed models could lead to a better solution. The methods given by Gitinavard and Shirazi^81^ and Mishra et al.^36^ does not consider this concept
Time complexity	Time complexity is associated to the computational size of model. The methods given by Gitinavard and Shirazi^81^ and Mishra et al.^36^ have less time complexity than developed model, because estimating the weights of criteria, DMEs’ weights, and considering the AOs in the procedure of the developed IF-MEREC-SWARA-ARAS tool increase the size of essential computations

### Discussion and implications of this work

The proposed ARAS method is extremely dependent on the knowledge of DMEs and the way of their decisions. As an efficient and simple MCDM process, it can efficiently obtain the optimum options on IFSs setting. The results of this study conclude that the suggested framework is unique with its integration of the fairly aggregation operator, IF-MEREC-SWARA and ARAS models on IFSs setting. Its effectiveness and feasibility are illustrated in terms of implementation on a case study of SIBOs selection.

The combination of the MEREC and the SWARA is an effective and relatively latest procedure for the estimation of integrated weights in MCDM problems. It has lower evaluation complexity than some different tools namely AHP, BWM, and other weighting tools. The key advantage of the developed tool is its capability to treat the subjective assessment of DMEs and obtain quantitative significance values to define the RD of each indicator. Various scholars claim that the wide-range implementation of the SWARA tool can be recognized to its mobility and user-friendliness as well as the prospect of uniting with extant models. The ARAS is a comparatively efficient tool for obtaining the solutions in complex problems, specifically those that are related with a variety of assessment problems on under subjective estimations. It relates the UD for obtaining the OPRs of SIBO options (Zavadskas and Turskis, 2010).

**Figure 4:**
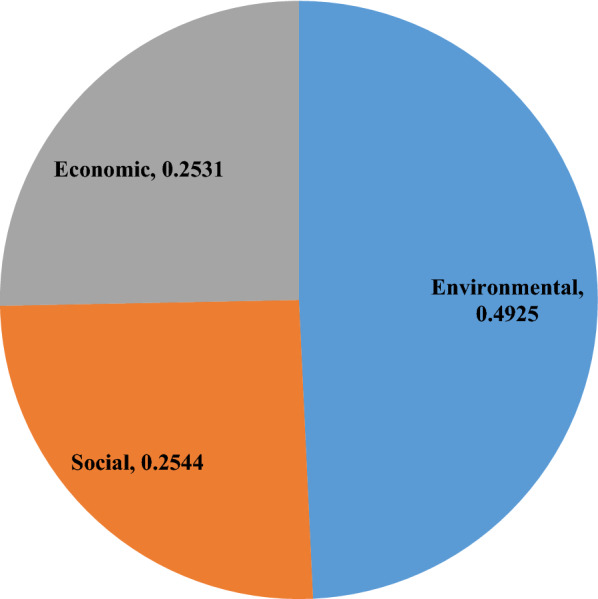
Relative weights of dimension of SDIs for SIBO selection.

The outcomes of the study show that environmental (EN) and social aspects are the two most significant dimensions with RDs of 0.4925 and 0.2544, respectively. The economical aspect with relative weights of 0.2531is the least significant one. The relative weights of different aspects are presented in Fig. 4.

**Figure 5:**
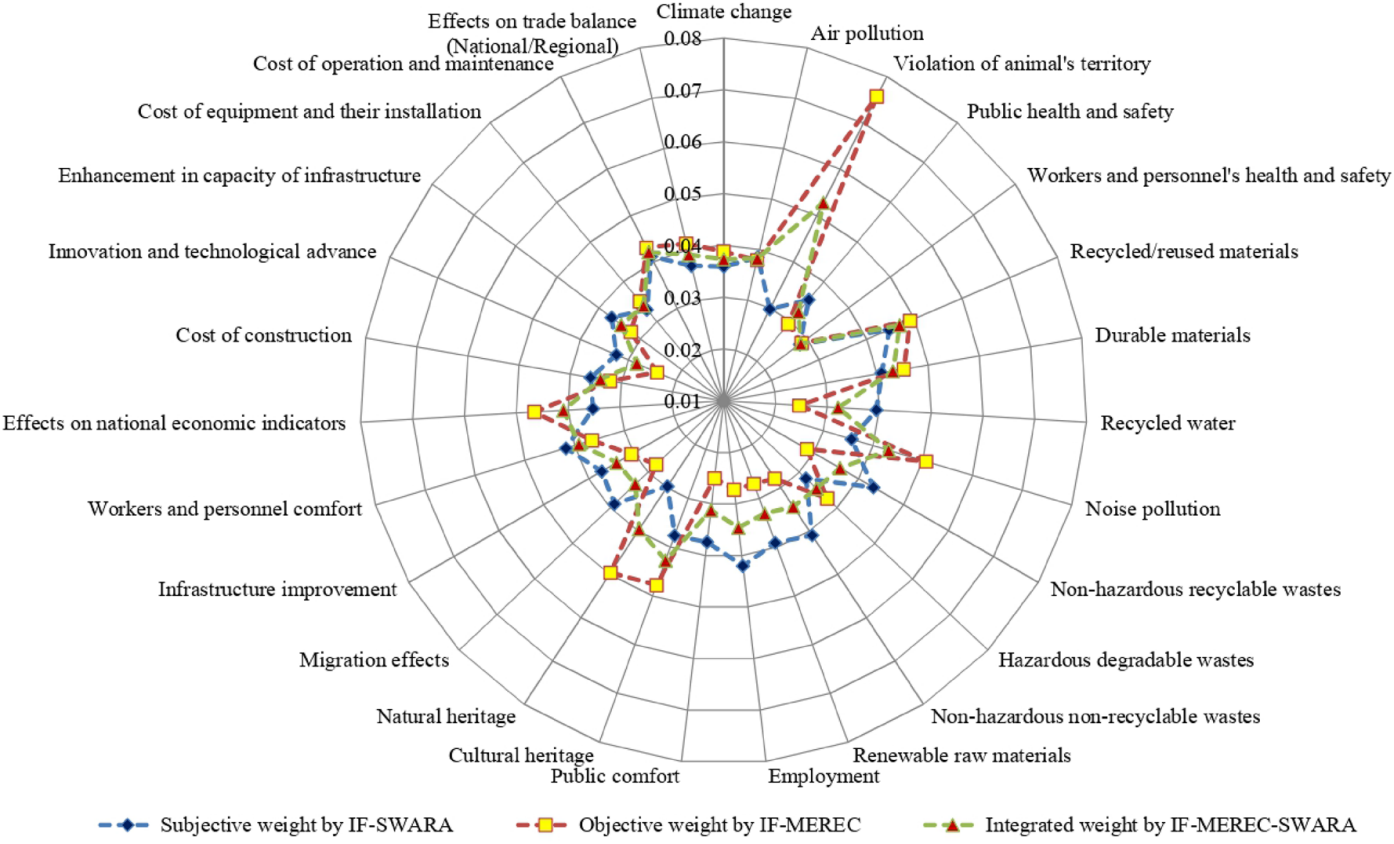
Preference order of SDIs using IF-MEREC-SWARA method.

As can be discussed in Fig. 5 and Table 8, based on the DMEs’ evaluations, *U*_3_ (Violation of animal's territory, 0.0528), *U*_6_ (Recycled/reused materials, 0.0469) and *U*_9_ (Noise pollution, 0.432) are the most important and *U*_23_ (Innovation and technological advance, 0.0282), *U*_5_ (Workers and personnel's health and safety, 0.0285) and *U*_15_ (Public comfort, 0.0312) are the least important SDIs of overall sustainability aspect. In environmental aspects, we find that *U*_3_ (Violation of animal's territory, 0.0528), *U*_6_ (Recycled/reused materials, 0.0469) have more significance than the other SDIs. In the social (SC) aspect, we can say that *U*_16_ (Cultural heritage, 0.0427) and *U*_17_ (natural heritage, 0.0396) have more significance than the other SDIs. Also, we can observe the maximum weights of *U*_26_ (Cost of operation and maintenance, 0.0412) and *U*_21_ (Effects on national economic indicators, 0.0409) in the economic (EC), which shows the higher important that the other SDIs.

Lastly, it should be revealed that if more projects even in small cities were planned as the case study, the outcomes, of course, might be reformed in comparison to projects placed in big cities, for instance, when it is planned to build a project in a small city, a project gains more social worth. The reason is that more social welfares would be brought to the public of a small city than a larger city in terms of job creation and infrastructure development. This indicates the assessment of such a project over others when more attention is waged to the social MCDM.

### Sensitivity analysis

**Figure 6:**
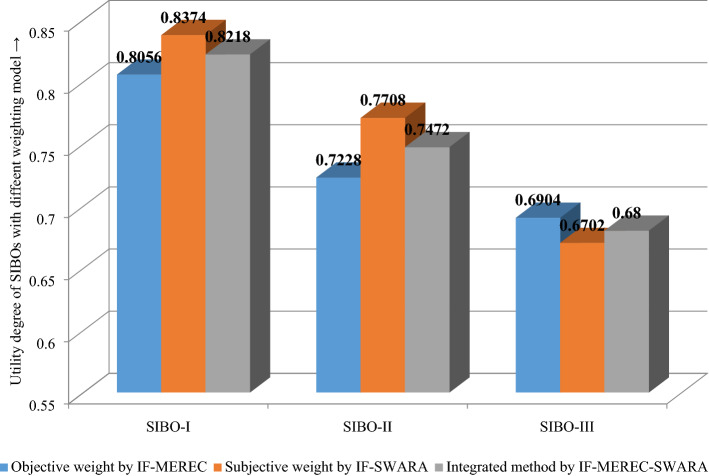
Sensitivity analysis for prioritizing SIBOs with different weighting procedures.

In this portion, we discuss the variation of weights of indicators from objective and subjective weights in the “IF-MEREC-SWARA method” for prioritizing SIBOs. In this line, the prioritizations of SIBOs have been obtained using the objective and subjective weights of indicators in lieu of IF-MEREC-SWARA model and are given in Table 18 and Fig. 6. From IF-MEREC, the UDs and priority of options are given as follows: The UDs of options are as *M*_1_ = 0*.*8056, *M*_2_ = 0*.*7228 and *M*_5_ = 0*.*6904 and prioritization of SIBOs is given as $M_{1} \succ M_{2} \succ M_{3} .$ Applying the IF-SWARA method, the UDs and priority of options are discussed as follows: The UD of each option as *M*_1_ = 0*.*8374, *M*_2_ = 0*.*7708 and *M*_3_ = 0*.*6702 and the ranks of SIBOs is given as $M_{1} \succ M_{2} \succ M_{3} .$ From aforesaid investigation, it is concluded that the utilization of diverse values of strategic coefficient will enhance the permanence of the IF-MEREC-SWARA-ARAS method.

**Table 18:** The UDs for prioritizing SIBOs over different weighting procedures.

Weighting model	UDs for SIBOs assessment			Ranks
	*M*_1_	*M*_2_	*M*_3_	
Objective weight by IF-MEREC	0.8056	0.7228	0.6904	$M_{1} \succ M_{2} \succ M_{3}$
Subjective weight by IF-SWARA	0.8374	0.7708	0.6702	$M_{1} \succ M_{2} \succ M_{3}$
Integrated method by IF-MEREC-SWARA	0.8218	0.7472	0.6800	$M_{1} \succ M_{2} \succ M_{3}$

## Conclusions

The aim of this study is to recommend a new MCDM tool for choosing the most suitable SIBO in uncertain environment. The primary contributions of this study are as follows:

- A novel intuitionistic fuzzy weighted fairly AOs and their properties are discussed, which overcome the drawback of existing intuitionistic fuzzy AOs.
- This paper further developed a methodology by integrating the fairly AO, the MEREC, the SWARA and the ARAS frameworks with IFSs. In the proposed methodology, the fairly AO has been utilized to aggregate the decision information. Moreover, the IF-MEREC-SWARA model has been used to determine the objective and subjective weights of the criteria from intuitionistic fuzzy perspective, while integrated ARAS method has developed to prioritize the alternatives by means of multiple criteria and uncertainty.
- A case study for SIBOs assessment has been presented to show the practicability of the present ARAS methodology. Comparative analysis has been discussed to confirm the robustness of the results acquired by proposed hybrid model. The main benefits of the presented framework are the ease of computation in intuitionistic fuzzy background and utilizing a model for deriving more reasonable weights of SDIs.

However, the proposed study needs to consider the technological and risk aspects of sustainability during the assessment of SIBOs. In addition, there is a need to consider the relation between SDIs, which is missing in the present work. More DMEs should be included in the assessment of SIBOs. In future, the developed methodology can be extended to different uncertain environments such as “q-rung orthopair fuzzy soft rough sets (q-ROFSRSs)”, “interval-valued Fermatean fuzzy sets”, “interval-valued hesitant Fermatean fuzzy sets (IVHFFSs)” etc. Moreover, the developed method can also be utilized in treating with diverse MCDM concerns, namely IoT smart city development, low-carbon supplier selection and project selection, and others.

## Data availibility

All data generated or analyzed during this study are included in this published article.
